# The effect of COVID-19 vaccination on change in contact and implications for transmission

**DOI:** 10.1016/j.epidem.2025.100827

**Published:** 2025-04-09

**Authors:** Carol Y. Liu, Aaron Siegler, Patrick Sullivan, Samuel M. Jenness, Stefan Flasche, Benjamin Lopman, Kristin Nelson

**Affiliations:** a Department of Epidemiology, Emory University Rollins School of Public Health, Atlanta, GA, USA; b Centre for Mathematical Modelling of Infectious Diseases, London School of Hygiene and Tropical Medicine, London, UK

**Keywords:** Behavior change during outbreaks, Transmission, COVID-19

## Abstract

**Background:**

Monitoring human behavior as epidemic intelligence can critically complement traditional surveillance systems during epidemics. Retrospective analysis of novel behavioral data streams initiated during the COVID-19 pandemic help illustrate their utility. During the pandemic, behavior changed rapidly and was increasingly influenced by individual choice in response to changes such as newly available vaccines. Vaccines provided substantial protection against severe disease and deaths; however, their effect on behavior is understudied and it is unclear if vaccine effects against infection fully offset relaxation of social distancing behaviors.

**Methods & results:**

We analyzed data from a longitudinal cohort sampled from U.S. households that measured contact rates, risk mitigation and COVID-19 vaccination status between August 2020-April 2022. Contact rates universally increased across survey rounds among all sociodemographic groups, but unvaccinated individuals had persistently higher contact rates. Using a multilevel generalized linear mixed effects model, we found that individuals who newly completed a primary vaccine series had an additional increase of 1.93 (95 % CI: 0.27–3.59) contacts compared to individuals who remained unvaccinated. Using observed contact rates to estimate transmission, we found that observed increases in contact rates were not fully offset by vaccine protection against infection, but transmission was still maintained below levels without distancing and vaccination despite clusters of individuals with high contact and no vaccination.

**Conclusion:**

We estimated changes in contact rates following vaccination and inferred the joint effect of changes in vaccination and contacts on population-level transmission, finding that observed increases in contact rates were not fully offset by vaccine effects. Our work highlights the potential utility of ongoing longitudinal monitoring of contact patterns during epidemics.

## 1. Introduction

Monitoring human behavior during epidemics can complement traditional surveillance systems during public health emergencies. Traditional surveillance methods focus on tracking the spread of disease through clinical and laboratory data but may not provide a complete picture of transmission, which relies primarily on human behavioral patterns. For instance, patterns of social interactions, adherence to mask-wearing and the uptake of vaccination all influence how a disease spreads through a population. During the COVID-19 pandemic, novel data streams on behavior, such as surveys on contact rates, generated real-time insights into changes to population-level social interactions, providing an opportunity to explore the causes of these changes, and their subsequent impact on epidemic trajectory.

The COVID-19 pandemic drastically altered contact patterns (Nelson and Lopman, 2022). Early in the pandemic, efforts to reduce transmission (e,g., shelter-in-place policies; and closures of schools, workplaces and public locations) centered on dramatic reductions in person-to-person interactions (Liu et al., 2021). As initial measures were relaxed, rates of contact gradually rebounded and began to fluctuate, increasingly shaped by individual choice rather than policy mandates (Coletti et al., 2020; Gimma et al., 2022a). In January 2021, the widespread rollout of COVID-19 vaccinations marked a new phase in the control of COVID-19. COVID-19 vaccinations substantially reduced morbidity and mortality (Watson et al., 2022; Koelle et al., 2022; Telenti et al., 2021) and permitted the easing of the most stringent social distancing policies.

As vaccination efforts intensified, speculation emerged on whether protection from vaccines would sufficiently offset potential increases in transmission following a return to normal social interactions (Trogen and Caplan, 2021). Individuals likely weighed the benefits of relaxed social distancing against the perceived and real risk of infection, severe illness and onward transmission after vaccination (Trogen and Caplan, 2021). Notably, risk mitigation tended to cluster within individuals. Individuals who adopted one mitigation measure were more likely to adopt multiple measures (Latkin et al., 2021; Rane et al., 2022; King et al., 2021), partitioning the population into highly protected groups who adopted multiple measures and highly exposed groups who adopted few or no measures (Arnold, 2023). Intense transmission within highly exposed subgroups can impact population-level transmission rates, and overall epidemic trajectory.

Contact rates in populations provide a quantifiable link between shifting individual behavior and population-level transmission. R_0_, the number of secondary infections generated by an average infectious individual in a fully susceptible population, is a function of both the number of contacts made by individuals and their level of susceptibility against infection. Although vaccines provided strong protection against severe SARS-CoV-2 infection, vaccine protection against mild infection and onward transmission is incomplete (Clifford et al., 2023). At the population-level, incomplete vaccine protection against infection followed by increased contact rates post-vaccination could lead to higher incidence than pre-vaccination (Trogen and Caplan, 2021). Understanding potentially counterintuitive population-level effects of vaccination, whereby the introduction of vaccination inadvertently increases disease incidence, is critical to understanding the overall health impact of a vaccine program in the context of a protracted pandemic.

There is a lack of evidence on the effect of receiving a COVID-19 vaccination on changes to individual-level contact rates in the U.S. In addition, the potential impact of tradeoffs between introducing vaccines and the resulting behavior changes on population-level transmission remains unquantified. Our analysis leverages longitudinal data obtained from a diverse U.S. cohort that spans the duration of vaccine rollout from August 2020 to March 2022. We assess the impact of changing individual-level vaccination on changes to an individual’s contact rate. We further estimate the impact of the joint effect of contact rates, vaccination coverage and vaccine protection on population-level transmission using a mathematical formulation that estimates the reproduction number, R.

## 2. Methods

### 2.1. Sampling

The COVIDVu study is a longitudinal survey that was conducted during the COVID-19 pandemic, consisting of a diverse and geographically representative cohort of individuals sampled from households in the United States. The address-based household sampling frame was previously described (Siegler et al., 2020). In brief, residential addresses were chosen to be representative of the U.S. population in terms of age, gender, race/ethnicity, education level, household income, region of residence and home ownership. From each household, a single household member > 18 years of age was randomly chosen to participate in the study. Surveys were conducted at four time points representing distinct periods of the COVID-19 pandemic in the U.S.: 1) August–December, 2020, during initial relaxation of the most stringent restrictions followed by a rise in cases in the winter of 2020; 2) March–April, 2021; during the start of widespread COVID-19 vaccine availability and a fall in cases 3) July–August, 2021; during continued relaxations in policies and case surges in the Delta wave and 4) March–April, 2022; shortly after the Omicron wave (Murray, 2022). The COVIDVu study was approved by the Emory University Institutional Review Board (STUDY00000695).

### 2.2. Survey data and latent class model to classify risk tolerance at baseline

Participants completed an online survey which included a set of questions that measured vaccination status (Siegler et al., 2020), risk mitigation behaviors, level of concern for new variants during the four survey periods, and included a contact survey adapted from previously published contact studies (Wong et al., 2022; Mossong et al., 2008; Gimma et al., 2022b; Kiti et al., 2021). Participants reported on the number of contacts they had the day before the survey by age of contact (0–4 years, 5–9 years, 10–19 years, 20–39 years, 40–59 years, 60–69 years, and 70 years and older) and by location (home, work, school, other locations). Contacts were classified as physical (physical touch, such as hug or kiss) or non-physical (being within 6 feet with an exchange of three or more words or for longer than 15 minutes) (Nelson et al., 2021; Mossong et al., 2008; Hoang et al., 2019; Melegaro et al., 2017; Hens et al., 2009; Kiti et al., 2014; Leung et al., 2017). Other information collected as part of the survey included sociodemographic characteristics (age, gender, race/ethnicity, household size, occupation status), presence of comorbidities and political affiliation at baseline. Age, gender and race/ethnicity were imputed when missing using hierarchical hot deck imputation where missing data was replaced with observed values from respondents with similar characteristics (Sullivan et al., 2021; Andridge and Little, 2010). The imputation preserved the distribution of the data while accounting for the hierarchical structure of the dataset. Data and code used for this analysis can be found in https://github.com/cliu822/contchng_vaxeffect.

We conducted latent class analysis (LCA) to classify participants into unobserved groups of similar patterns of intrinsic risk tolerance. We used participant-reported level of adoption of various risk mitigation behaviors at baseline as indicator inputs into the LCA. Individuals were assigned to latent classes based on the probabilities of belonging to each class based on the model of choice using R software package “poLCA” (Linzer and Lewis, 2011) and LCA classes were considered as model covariates. (Methods in SI.2.)

### 2.3. Modeling effect of vaccination on contact rate

We fit a multivariate mixed linear regression to estimate the effect of vaccination on change in contact rate, with a random intercept for the individual to account for repeated survey responses from the same participants. The primary outcome was change in number of contacts made in one day between two consecutive periods of data collection, chosen to examine how contacts evolved over time and to isolate the effect of vaccination from broader temporal trends in contact rates. Secondary outcomes were changes in location-specific contacts between two consecutive periods made at work, home and at other locations (i.e., stores and restaurants, public transit, gym). Similar to previous studies (Wambua et al., 2023; Wong et al., 2023), to reduce the effect of outliers and potentially unrealistic survey responses, location-specific contact rates were capped at the 99th percentile of reported contact rates. Briefly, contact rates exceeding the 99th percentile of the distribution were coded as the value at the 99th percentile.

The primary exposure was change in vaccination status: 1) unvaccinated at current period (no change); 2) one SARS-CoV-2 vaccine dose between previous period and current period; 3) completed primary series between previous period and current period; 4) completed second dose between previous period and current period and 5) fully vaccinated before current period (no change) (schematic in SI.3). The secondary exposure was vaccination coverage in the participant’s county of residence at the time of survey completion. We decided *a priori* to adjust for age group and household size, which are known to influence both contact and COVID-19 vaccine uptake (Bilinski et al., 2021; Yan et al., 2021). Since contact rates fluctuated during the pandemic in response to both policy measures and individual risk perception (Wambua et al., 2023; Wong et al., 2023), we decided *a priori* to adjust for time-varying covariates of state-wide COVID-19 stringency level, using the Oxford Stringency Index (OSI) (Hale et al., 2020), and the level of changing personal concern for new variants. We conducted stepwise backwards selection to decide on the most parsimonious set of additional covariates (gender, race/ethnicity, self-reported political affiliation, income status, employment status, comorbidity, baseline LCA) to include in the fully adjusted multivariate model. Variables were eliminated based on change-in-estimate criterion. At each step, the variable producing the least amount of change in the effect estimate when removed was removed until further removal caused a change exceeding 10 %.

### 2.4. Estimating the impact of contact change on transmission potential

We incorporated both vaccine effectiveness and changing contact rates among vaccinated and unvaccinated participants into a mathematical framework to estimate their joint effects on transmission using the Next Generation Matrix (NGM) at each round t. The NGM quantifies the number of secondary infections generated in each population subgroup based on heterogeneous mixing patterns between and within subgroup. Here, we stratify the population into vaccinated and unvaccinated subgroups (Eq. 1). Briefly, $R_{vv}$ is the number of secondary infections generated between vaccinated persons interacting with other vaccinated persons. Under assumptions of proportional mixing between the two subgroups based on the vaccine coverage in the US at the time of each survey, $R_{vv}$ is defined by $c_{v,t}$, contact rate among vaccinated persons in data collection round t; $\chi_{t}$, vaccine coverage; β, the probability of transmission between two unvaccinated individuals; $VE_{s}$, vaccine effectiveness against susceptibility (50 % for main analysis) and d, the duration of infection (7 days) (Diekmann et al., 2010). We estimate β through the formula $\beta =\frac{R}{d*c}$, assuming an initial reproduction number, R, of 3 (Dhungel et al., 2022; Read et al., 2020; Billah et al., 2020) and a daily mean contact rate of 16 in the U.S. under no social distancing (Prem et al., 2017). $R_{round}$ is estimated by solving for the dominant eigenvalue of the NGM (Gimma et al., 2022b; Diekmann et al., 2010; Tomori et al., 2021) (Eq. 2). We then produce NGMs accounting for heterogeneity in local coverage and a range of mixing assortativity where vaccinated individuals preferentially mix with other vaccinated individuals and unvaccinated with other unvaccinated.


(1) $$NGM_{t}=\left(\begin{array}{cc} R_{vv} & R_{vu}\\ R_{uv} & R_{uu} \end{array}\right)=\left(\begin{array}{cc} c_{v,t}\chi_{t}\beta (1-VE_{s})d & c_{v,t}(1-\chi_{t})\beta d\\ c_{u,t}\chi_{t}\beta (1-VE_{s})d & c_{u,t}(1-\chi_{t})\beta d \end{array}\right)$$

(2) $$R_{round}=\lambda (NGM_{t})$$

## 3. Results

### 3.1. Participant description

A total of 2403 adult participants aged 18 years and above completed all four survey rounds and were included in the analysis. Among the included participants, the median age was 52 years (IQR: 36–65) at baseline and 1496 (62 %) were female. Most identified as non-Hispanic White (n = 1657, 69 %), followed by non-Hispanic Black (n = 302, 13 %), Hispanic (n = 276, 11 %), non-Hispanic Asian (n = 126, 5 %) and non-Hispanic Other (n = 42, 2 %), comparable to the distribution of race and ethnicity in the U.S (US population by year, race, age, ethnicity, & more, 2023).

### 3.2. Latent Class Analysis of risk mitigation measures to classify risk tolerance

We included all available variables on risk mitigation behavior into the latent class classification (SI.2). Model diagnostic criteria were met for 3- and 4- class solutions (SI.2.5) and we decided to use a 4-class solution for increased distinguishing power offered by more classes. In the 4-class solution, individuals classified into the lowest risk tolerance group were substantially more likely to engage in risk mitigation behavior. For example, 90 % of individuals with the lowest risk tolerance reportedly always wore a mask when going out compared to 8 % of individuals with the highest risk tolerance (SI.2.6). Individuals with the lowest risk tolerance at baseline were less likely to remain unvaccinated although Spearman’s rank correlation coefficient showed only a weak correlation between vaccination status and risk tolerance classification (−0.16 on a scale of −1–1 where 0 is no correlation) (SI.4).

### 3.3. Change in contact rates over time

Overall, the mean number of total daily contacts increased across survey rounds, from 8.4 (95 % CI: 7.8–9.0) at baseline, 9.8 (95 % CI: 9.1–10.6) at round 2, 11.7 (95 % CI: 10.8–12.8) at round 3 and 14.7 (95 % CI: 13.7–15.8) at round 4 (Table 1). At baseline, mean numbers of contacts reported by participants varied by locations: 4.0 (95 % CI: 3.4–4.5), 2.4 (95 % CI: 2.2–2.6), 1.9 (95 % CI: 1.8–2.0) and 0.1 (95 % CI: 0.1–0.1) contacts at work, other locations, home, and school, respectively, accounting for 48 %, 29 %, 22 % and 1 % of all contacts, respectively. Mean contact rates increased at both work and other locations throughout the survey rounds but remained similar at home and at school (Table 1).

### 3.4. Vaccination and contact rates

**Fig. 1.**
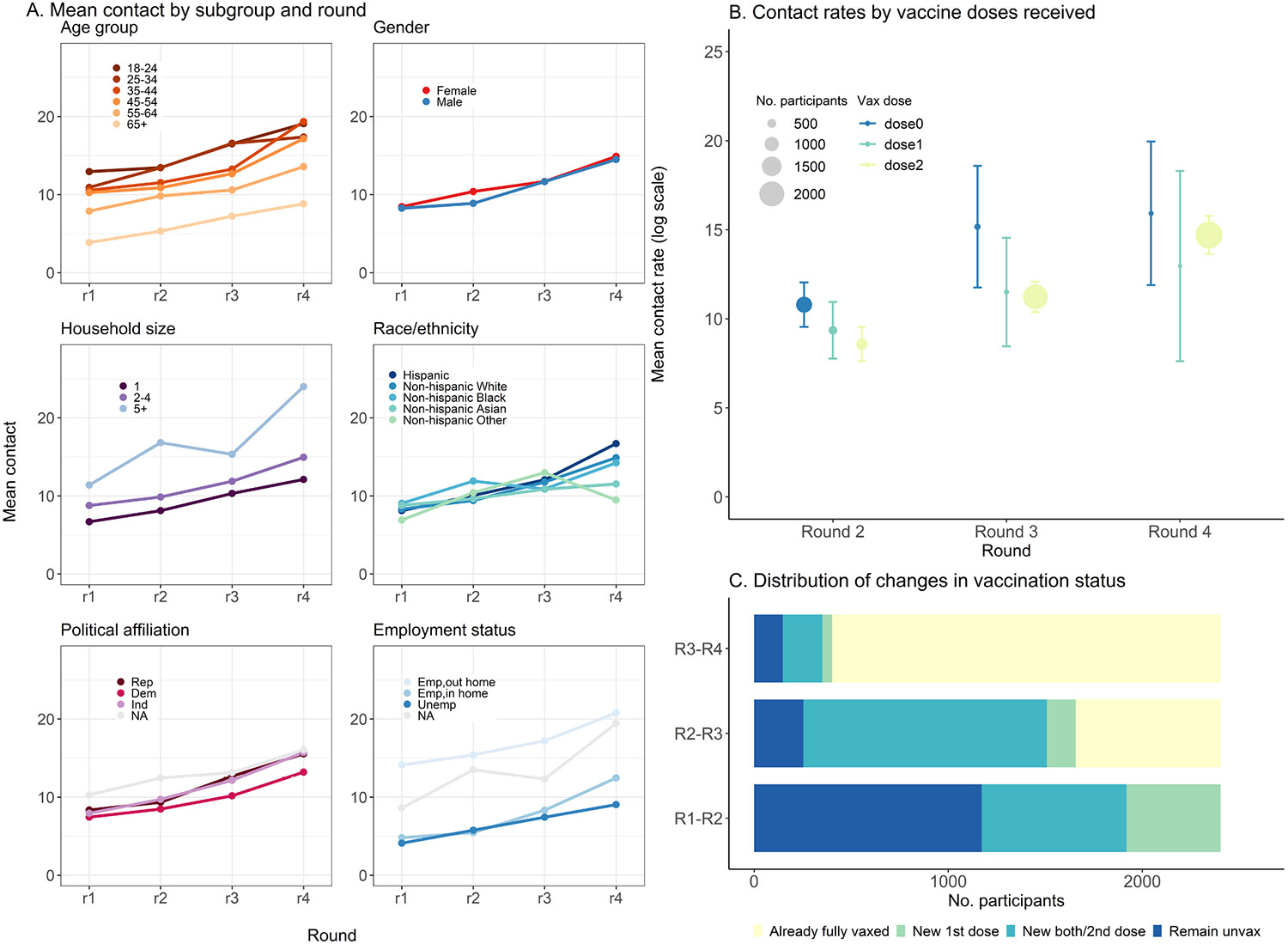
Distribution of mean contact rates and exposure over survey round. A. Distribution of mean contact over survey round stratified by age group, gender, race/ethnicity, household size, political affiliation, and employment status. B. Mean contact rates by vaccine doses (0-blue, 1-green, 2-yellow) received for each round with the size of circle representing the number of participants reporting each vaccination status in each round. C. Distribution of the main exposure, change in vaccination status, over round of survey.

COVID-19 vaccinations became available for the general population during round 2, and vaccination rates among participants increased between round 2 and round 4. In round 2, 1773 (49 %) of participants were unvaccinated and by round 4, only 255 (11 %) remained unvaccinated. In each round, contact rates were higher among participants remaining unvaccinated compared to those who had completed the primary series (10.8 contacts (95 % CI: 9.5–12.0) versus 8.6 contacts (95 % CI: 7.6–9.5 in round 2 and 15.2 contacts (95 % CI: 11.7–18.6) versus11.2 contacts (95 % CI: 10.4–12.1) in round 3) (Fig. 1; SI.6). The overall primary series vaccination coverage among all age groups in the U.S. rose from 11.5 % in round 2–65.9 % by round 4 (Tomori et al., 2021). Contact rates were comparable across counties with different levels of vaccination coverage in rounds 2 and 3 but participants residing in counties with higher coverage had lower contact rates compared to those residing in counties with lower coverage (SI.6).

### 3.5. Variation in contact rates by key covariates

At baseline, contact rates differed by age group, employment status, risk tolerance, presence of comorbidities and household size and were comparable by gender, household income, race/ethnicity, and political affiliation. Younger individuals had higher contact rates, with 18–24-year-olds reporting the most contacts at 12.9 (95 % CI: 9.1–16.8). Employed individuals required to work outside of their homes reported the most contacts at 14.1 (95 % CI: 12.8–15.5). Individuals classified as having high risk tolerance had the highest contacts at 14 (95 % CI: 11.4–16.7) and those classified as medium-low risk tolerance had the lowest contacts at 4.2 (95 % CI: 3.7–4.7). Individuals without comorbidities had more contacts (9.1; 95 % CI: 8.2–10.0) than those with at least one comorbidity (7.7; 95 % CI: 6.9–8.5). (Table 1).

Contacts increased in all subgroups through survey rounds across almost all sociodemographic groups. Absolute increases in contact between rounds one and four were comparable across age group, gender, comorbidity status and political affiliation. For example, mean contacts among 18–24-year-olds increased by 6.2 contacts between rounds one and four, comparable to an increase of 5.0 contacts among 65 + year olds.

Contact rates further differed by time-varying covariates of self-reported concern over new variants and stringency of state-level COVID-19 mitigation policy. Over survey round, participants reported decreased concern over new variants and were less likely to live in states with stringent COVID-19 mitigation policies such as restrictions to public gatherings and school closures. Participants who reported increased concern over new variants and participants who resided in states with more stringent mitigation policies reported fewer contacts (SI.6).

### 3.6. Change in individual-level vaccination status over time

We categorized our main exposure as the change in vaccination status between rounds to isolate the effect of receiving vaccination on change in contact behavior. Between round 1 and 2, 1173 (49 %) remained unvaccinated, 484 (20 %) newly received the first dose and 746 (31 %) newly completed the primary series. Between round 2 and 3, 254 (11 %) remained unvaccinated, 149 (6 %) newly received the first dose, 1254 (53 %) newly completed the primary series. Between round 3 and 4, 148 (6 %) remained unvaccinated, 50 (2 %) newly received the first dose, 204 (9 %) newly completed the primary series and 2001 (83 %) were already fully vaccinated before round 3 (Fig. 1C).

### 3.7. Effect of vaccination on change in contact rates

**Fig. 2.**
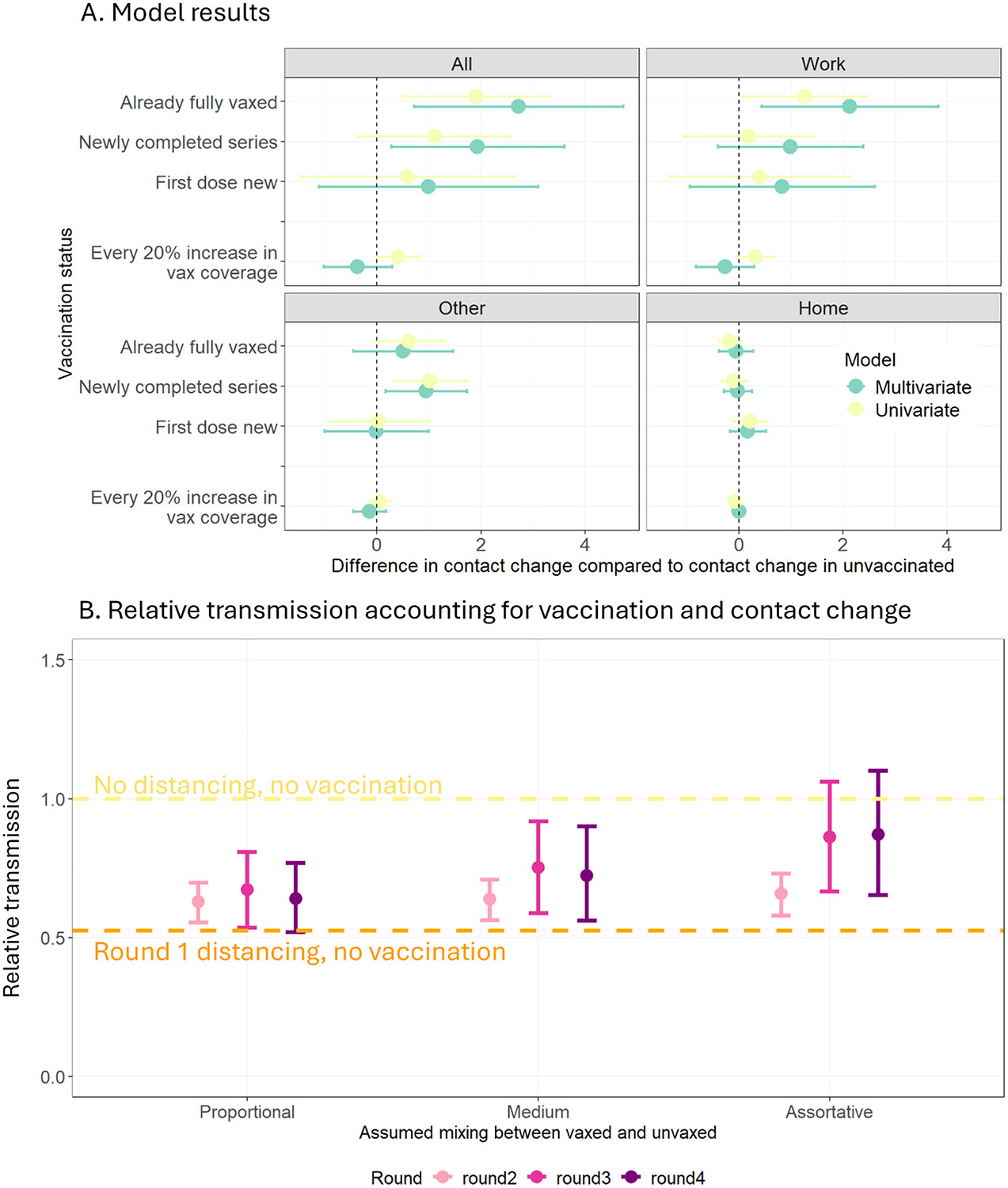
Plots of main effect estimate and estimated relative transmission based on changing contact rates and vaccination A. Plots of difference in contact change among vaccinated groups compared to contact change among unvaccinated and the effect estimate for every 20 % increase in county-level vaccination coverage for multivariate (blue) and univariate (yellow) estimates among contacts in all locations, at work, at other leisure locations and at home. B. Estimated relative transmission based on measured contact among vaccinated and unvaccinated individuals for each round using a vaccine effectiveness against infection of 50 % and varying assortativity assumption from proportional to assortative (unvaccinated individuals only contact other unvaccinated individuals.

After stepwise backwards regression, we arrived at a multivariate model that was adjusted for age group, household size, political affiliation, employment status, risk tolerance at baseline estimated by a Latent Class Analysis, baseline contact rates, change in concern over pandemic and stringency of COVID-19 policy at the state-level (OSI). Our model outcome is change in contact rates between consecutive survey rounds, chosen to explore marginal differences in increases due to vaccination in the context of universally increasing contact rates. In the fully adjusted model, individuals who completed a primary series between two survey rounds increased their contacts by an additional 1.93 (95 % CI: 0.27–3.59) contacts compared to individuals who remained unvaccinated. Individuals already fully vaccinated had an additional increase of 2.72 (95 %CI: 0.71–4.73) contacts, and individuals who newly received the first vaccine dose had a slight increase of contacts 0.99 (95 %CI: −1.12–3.1) (Fig. 2; Table 2). Multivariate models using change in location-specific contacts at work, other leisure locations and home as the outcome showed that individual-level vaccination status affected contacts at work and at other locations and did not affect contacts at home. Individuals newly completing a primary series reported additional increases of 0.99 (95 % CI: −0.4–2.39), 0.63 (95 % CI: −0.15–1.51) and 0.94 (95 % CI: 0.16–1.73) contacts at work, other locations, and home, respectively. We did not find evidence that increasing vaccination coverage in participants’ county of residence was associated with a change in contact.

### 3.8. Impact of differential changes in contact rates among vaccinated and unvaccinated on transmission

In the context of increasing vaccine coverage and increasing contacts following vaccination, we estimate their joint effects on potential transmission using the NGM. We found that using observed increases in contact rates and assuming up to 50 % vaccine effectiveness against infection, $R_{\text{round}}$ estimates from rounds 2–4 following vaccine introduction were lower than the assumed R_0_ (no vaccination and no distancing) but were higher than R estimates from round 1 (no vaccination and more stringent distancing). These results suggest that vaccine protection against infection was unable to fully offset observed increases in contact rates with respect to transmission. However, despite increases in contact rates among unvaccinated individuals, post-vaccination $R_{\text{round}}$ estimates did not exceed R_0_ even under assumptions of highly assortative mixing where unvaccinated individuals exclusively contact other unvaccinated individuals (Fig. 2).

## 4. Discussion

Using longitudinal data from the U.S. spanning 18 months of the COVID-19 pandemic, we found that while unvaccinated individuals persistently reported the highest contact rates, those newly completing their primary series had a greater increase in contact compared to unvaccinated individuals. Further, individual-level vaccination had more impact on changes in contact rates at work and at other locations than at home. We further show that while vaccination is unable to fully offset observed increases in contact with respect to transmission, the extent of contact increase following vaccination is unlikely to raise population-level transmission to above that of pre-social distancing levels, even with highly preferential mixing among unvaccinated individuals.

Similar to previous studies conducted both during (Liu et al., 2021; Gimma et al., 2022b; Nelson et al., 2021; Wong et al., 2023; Andrejko et al., 2022; Chin et al., 2021; Feehan and Mahmud, 2021; Salomon et al., 2021; Dorelien et al., 2020) and before the pandemic, contact rates differed by age group, employment status, risk tolerance, presence of comorbidities and household size (Mossong et al., 2008; Mousa et al., 2021). Despite these differences, contact rates universally increased across most sociodemographic groups during the study period. Our findings of additional increase in contact among those newly completing a primary vaccine series complements evidence from the U.S (Jia et al., 2022)., Japan (Yamamura et al., 2022), Italy (Corea et al., 2022), Bangladesh (Hossain et al., 2022), Israel (Rahamim-Cohen et al., 2021), and Brazil (Mello López et al., 2022) that observed a decline in protective behaviors among vaccinated individuals. While these studies assessed the adoption of risk mitigation behavior post-vaccination (Calamari et al., 2022), we focused on quantifying the effects on contact rates, metrics that can be directly incorporated into mathematical frameworks to estimate transmission intensity. Our findings further isolated the effect of vaccination on individual-level contact changes by comparing contact changes among recently vaccinated individuals to those who remained unvaccinated in the same period. These estimates extend evidence from a multi-country European study that found increased contact numbers among vaccinated individuals compared to unvaccinated individuals (Wambua et al., 2023).

We use a simple mathematical framework to relate individual behavior changes to the expected population-level outcomes for COVID-19 transmission. Novel mathematical models have proposed to explicitly incorporate social and behavioral feedback loops into mathematical models where social interactions are modified based on equations dependent on contextual changes such as vaccination or an increase in incidence (Funk et al., 2010; Bedson et al., 2021; Cascante-Vega et al., 2022; Auld, 2003; Bergstrom and Hanage, 2024; Saad-Roy and Traulsen, 2023). These frameworks typically assume perfectly rational human behavior. Empirical data on the expected increase in contact rates from changes in individual vaccination status enables the explicit representation of their relationship in mathematical models. and lead to more realistic estimates of the population-level vaccine impact. Timely estimates that show increased transmission due to increases in contact rates despite the protective effects of vaccination could prompt public health messaging that discuss residual risks associated with widespread increases in social interactions following vaccination.

There are several limitations to this analysis. Baseline participation rates were 10–15 % which are low but typical for mailed surveys using address-based sampling frames (Fahimi et al., 2008). Because attrition between survey rounds was also likely differential, the subset of participants who responded to all four surveys is not rigorously representative of the U.S. population; however, we find similar distributions of key covariates among those initially enrolled and among those completing all survey rounds (SI.1). Our cohort had a higher rate of completing primary series of vaccination than the general U.S. population. We find that by round 4, 91 % of our cohort had completed the primary series compared to 79 % of the U.S. population aged 18 years and above. This suggests that our cohort has more health awareness, and results may not be generalizable to the larger U.S. population. Social desirability bias from self-report may result in underestimates of contact and overestimates to adherence of other risk mitigation measures; however, if underestimation was consistent across rounds, changes in contact rates would remain valid even if the absolute rates are underestimated. Many individuals received either their first or second dose of the vaccine between the second and third time points when a range of policies and sentiments regarding COVID-19 precautions were changing. Additional contemporaneous and unmeasured changes such as increasing population-level vaccine coverage and subsequent changes in remote work policies, could have affected changes in contact rates. Clustering of health-protective behaviors within close social networks may also act as an unmeasured confounder, because individuals in the same network may share similar vaccination decisions and contact behavior. In general, the relationship between recent vaccination and additional increases in contact rates is not synonymous with causation; however, we adjusted for numerous important factors that affect contact rates such as a metric describing stringency of state-level pandemic restrictions, baseline risk tolerance and concern for new variants. Lastly, our estimated $R_{\text{round}}$ values do not account for natural immunity and is meant to be interpreted as the relative transmission impact of changes in vaccination and in behavior alone.

## 5. Conclusion

In conclusion, our study highlights insights generated from the continuous monitoring of human behavior, particularly contact patterns, during the COVID-19 pandemic. Coupling behavioral insights with vaccination data allows contemporaneous estimation of population-level transmission and better-informed public health strategies. Our results emphasize the utility of ongoing behavioral surveillance to guide adaptive responses to evolving epidemic conditions.

## Supplementary Material

Supplementary material

## Figures and Tables

**Table 1:** Mean contact rate stratified by participant characteristics.

Variable	Value	Total (%) (N = 2403)	Mean contact rate (95 % CI)			
			Round 1	Round 2	Round 3	Round 4
			
			(Aug-Dec, 2020)	(Mar-Apr, 2021)	(July-Aug, 2021)	(Mar-April, 2022)
**Location-specific contacts among all participants**						
	Work	2403 (100 %)	4 (3.4–4.5)	4.8 (4.1–5.4)	5.3 (4.6–6)	7.5 (6.7–8.4)
	Other		2.4 (2.2–2.6)	2.7 (2.4–3)	4.1 (3.7–4.4)	4.4 (4–4.9)
	Home		1.9 (1.8–2)	2.2 (2.1–2.4)	2.3 (2.1–2.4)	2.3 (2.2–2.4)
	School		0.1 (0.1–0.1)	0.1 (0.1–0.1)	0.1 (0.1–0.1)	0.5 (0.4–0.6)
**All-location contacts stratified by population subgroup**						
**Overall**			**8.4 (7.8–9)**	**9.8 (9.1–10.6)**	**11.7 (10.8–12.5)**	**14.7 (13.7–15.8)**
**Age group**	18–24	111 (5 %)	12.9 (9.1–16.8)	13.4 (9.9–17)	16.5 (11–22)	19.1 (14.2–24)
	25–34	345 (14 %)	10.9 (9.1–12.8)	13.4 (10.9–15.9)	16.6 (13.6–19.6)	17.4 (14.5–20.2)
	35–44	407 (17 %)	10.6 (9–12.2)	11.5 (9.6–13.4)	13.3 (11.1–15.4)	19.3 (16.3–22.4)
	45–54	417 (17 %)	10.2 (8.5–12)	10.9 (9–12.8)	12.7 (10.7–14.7)	17.2 (14.4–19.9)
	55–64	502 (21 %)	7.9 (6.6–9.1)	9.8 (8–11.7)	10.6 (9–12.2)	13.6 (11.3–15.8)
	65 +	621 (26 %)	3.9 (3.3–4.4)	5.3 (4.6–6)	7.2 (6.3–8.1)	8.8 (7.7–10)
**Gender**	Female	1496 (62 %)	8.4 (7.7–9.2)	10.4 (9.4–11.4)	11.7 (10.6–12.7)	14.9 (13.6–16.2)
	Male	907 (38 %)	8.3 (7.3–9.2)	8.9 (7.8–10)	11.7 (10.4–12.9)	14.5 (12.9–16.1)
**Race/ethnicity**	Hispanic	276 (11 %)	8.1 (6.5–9.7)	10 (7.8–12.3)	12.1 (9.6–14.5)	16.7 (13.1–20.3)
	Non-Hispanic, White	1657 (69 %)	8.3 (7.6–9)	9.4 (8.6–10.2)	11.8 (10.8–12.8)	14.9 (13.7–16.1)
	Non-Hispanic, Black	302 (13 %)	9 (7–11.1)	11.9 (9.1–14.7)	10.9 (8.7–13.1)	14.2 (11–17.4)
	Non-Hispanic, Asian	126 (5 %)	8.8 (6.1–11.5)	9.6 (6.7–12.6)	10.8 (7.3–14.3)	11.5 (8.4–14.6)
	Non-Hispanic, Other	42 (2 %)	6.9 (3.6–10.2)	10.4 (3.7–17.1)	13 (5.3–20.6)	9.5 (5.3–13.6)
**Household size**	1	638 (27 %)	6.7 (5.6–7.8)	8.1 (6.7–9.5)	10.3 (8.8–11.8)	12.1 (10.3–13.9)
	2–4	1619 (67 %)	8.8 (8–9.5)	9.9 (9–10.7)	11.9 (10.9–12.9)	14.9 (13.8–16.1)
	5+	146 (6 %)	11.4 (8.7–14.1)	16.8 (12.3–21.3)	15.3 (11.3–19.4)	24 (17.3–30.7)
**Self-reported political affiliation**	Democratic	996 (41 %)	7.4 (6.6–8.3)	8.5 (7.5–9.5)	10.2 (9.1–11.3)	13.2 (11.7–14.8)
	Republican	378 (16 %)	8.4 (7–9.8)	9.3 (7.7–11)	12.7 (10.7–14.7)	15.5 (13.2–17.9)
	Independent	445 (19 %)	7.9 (6.6–9.3)	9.7 (8–11.5)	12.2 (10.1–14.2)	15.7 (13.2–18.2)
	Unknown	584 (24 %)	10.3 (8.9–11.7)	12.4 (10.6–14.3)	13.1 (11.2–15.1)	16.1 (13.9–18.3)
**Employment status**	Emp,in home	472 (20 %)	4.8 (4.1–5.5)	5.5 (4.6–6.4)	8.3 (6.8–9.9)	12.5 (10.2–14.7)
	Emp,out home	950 (40 %)	14.1 (12.8–15.5)	15.4 (13.9–17)	17.2 (15.6–18.9)	20.8 (18.8–22.8)
	Unemp	891 (37 %)	4.1 (3.7–4.5)	5.8 (5.1–6.5)	7.4 (6.6–8.2)	9 (8.1–10)
	Unknown	90 (4 %)	8.6 (6.3–10.9)	13.5 (7.7–19.3)	12.3 (7.5–17.1)	19.5 (12.8–26.2)
**Household income**	0-$24,999	250 (10 %)	8.6 (6.6–10.7)	11 (8.3–13.7)	10.4 (8–12.8)	14.1 (10.8–17.4)
	$25,000-$74,999	756 (31 %)	9 (7.8–10.2)	11.6 (9.9–13.2)	13.1 (11.3–14.8)	15.8 (13.8–17.8)
	$75,000-$149,999	695 (29 %)	8.3 (7.2–9.3)	8.7 (7.5–9.9)	10.9 (9.7–12.2)	14.2 (12.5–15.9)
	Greater than $150,000	384 (16 %)	8.4 (6.9–9.9)	9.3 (7.8–10.8)	12.4 (10.4–14.5)	15.5 (13–18)
	Unknown	318 (13 %)	6.8 (5.4–8.2)	7.7 (6.3–9.1)	10 (7.9–12)	12.9 (10.3–15.6)
**Comorbidities**	No	1151 (48 %)	9.1 (8.2–10)	10.3 (9.2–11.4)	12.4 (11.2–13.6)	15.7 (14.2–17.3)
	Yes	1252 (52 %)	7.7 (6.9–8.5)	9.4 (8.3–10.4)	11 (9.9–12.1)	13.8 (12.5–15.2)
**Risk tolerance**^a^ **(from Latent Class Analysis)**	High	208 (9 %)	14 (11.4–16.7)	18.2 (14.6–21.8)	20.8 (16.5–25.2)	24 (19.5–28.4)
	Med-high	841 (35 %)	9.1 (8.1–10)	9.7 (8.6–10.8)	11.2 (10–12.3)	14.9 (13.4–16.5)
	Med-low	856 (36 %)	4.2 (3.7–4.7)	6.6 (5.6–7.7)	8.3 (7.2–9.3)	10.9 (9.5–12.4)
	Low	498 (21 %)	12 (10.2–13.8)	12 (10.1–14)	14.5 (12.3–16.7)	17.1 (14.4–19.8)

a Risk tolerance characterized by latent class analysis of responses to a set of survey questions related to risk mitigation behavior

**Table 2:** Univariate and multivariate effect estimates of individual-level vaccination status and county-level vaccination status on change in contact rates.

Covariate	Category	Change in number of contacts	
		Univariate associations	Multivariate associations
**Intercept**			6.95 (1.65–12.25)
**Change in vaccination status**	Remain unvaxed		
	First dose new	0.59 (−1.46–2.64)	0.99 (−1.12–3.1)
	Newly completed series	1.11 (−0.37–2.58)	1.93 (0.27–3.59)
	Already fully vaxed	1.90 (0.48–3.31)	2.72 (0.71–4.73)
**Every 20 % increase in county-level vax coverage**		0.41 (−0.01–0.83)	−0.37 (−1.02–0.29)
**Age group**	18–24 yrs		
	25–34 yrs	0.10 (−2.71–2.92)	−0.06 (−2.98–2.86)
	35–44 yrs	0.88 (−1.88–3.65)	0.63 (−2.24–3.51)
	45–54 yrs	0.26 (−2.5–3.02)	0.42 (−2.47–3.31)
	55–64 yrs	−0.15 (−2.86–2.56)	−0.07 (−2.89–2.75)
	64 + yrs	−0.39 (−3.06–2.27)	−0.67 (−3.52–2.18)
**Gender**	Female		
	Male	−0.08 (−1.16–1.01)	
**Race ethnicity**	Hispanic		
	Non-Hispanic, White	−0.67 (−2.35–1.01)	
	Non-Hispanic, Black	−1.13 (−3.29–1.02)	
	Non-Hispanic, Asian	−1.95 (−4.72–0.83)	
	Non-Hispanic, Other	−2.01 (−6.28–2.27)	
**Household size**	1		
	2–4	0.25 (−0.95–1.46)	0.16 (−1.08–1.39)
	5 +	2.40 (0.03–4.77)	1.56 (−0.96–4.08)
**Political affiliation**	Dem		
	Rep	0.47 (−1.09–2.03)	−0.04 (−1.7–1.62)
	Ind	0.67 (−0.8–2.15)	0.68 (−0.81–2.18)
	Unknown	0.47 (−1.09–2.03)	−0.04 (−1.7–1.62)
**Employment status**	Emp,in home		
	Emp,out home	−0.33 (−1.78–1.13)	−0.39 (−1.9–1.12)
	Unemp	−0.91 (−2.38–0.56)	−0.67 (−2.33–0.99)
	Unknown	1.07 (−1.9–4.04)	0.43 (−2.59–3.46)
**Household income**	$0-$24,999		
	$25,000-$74,999	0.44 (−1.5–2.37)	
	$75,000-$149,999	0.16 (−1.8–2.12)	
	More than $150,000	0.55 (−1.61–2.71)	
**Comorbidities**	No		
	Yes	−0.18 (−1.23–0.88)	
**Risk tolerance (from Latent Class Analysis)**	High		
	Med-high	−1.35 (−3.35–0.65)	−1.28 (−3.4–0.84)
	Med-low	−1.08 (−3.08–0.92)	−0.94 (−3.12–1.25)
	Low	−1.61 (−3.74–0.53)	−1.61 (−3.86–0.64)
**Unit increase in baseline contact rates**		−0.16 (−0.2-−0.13)	
**Change in concern over pandemic**	Decreased greatly		
	Decreased slightly	−2.30 (−6.25–1.66)	−2.39 (−6.37–1.58)
	No change	−4.07 (−7.14-−1)	−3.83 (−6.93-−0.74)
	Increased slightly	−5.35 (−8.4-−2.3)	−5.06 (−8.19-−1.93)
	Increased greatly	−4.46 (−7.82-−1.11)	−4.25 (−7.7-−0.81)
**Unit increase in state-wide Oxford Stringency Index**		−0.04 (−0.08–0)	−0.01 (−0.07–0.04)

a Main effect is the difference in change in contact rates between rounds among individuals with various vaccination status (first dose new, both dose new, second dose new and already fully vaccinated) compared to the change in contact rates among unvaccinated individuals.

b The multivariate model adjusted for age group, household size, political affiliation, employment status, risk tolerance at baseline estimated by a Latent Class Analysis, change in concern over pandemic and the state-wide Oxford Stringency Index for state-level COVID-19 mitigation

## Data Availability

I have shared a link to Github repo with the data and code.
