# Supplementary material for: The effect of COVID-19 vaccination on change in contact and implications for transmission
Source: Epidemics. Author manuscript; Available in PMC 2025 Jun 16. (PMC12167678; doi:10.1016/j.epidem.2025.100827)
Supplement: Supplementary material [file NIHMS2080978-supplement-Supplementary_material.docx]

**Supplementary Information**

Table of Contents

[SI.1 Comparing distribution of covariates among initially enrolled study population and those completing follow-up 23](#_Toc172056999)

[SI.2 Additional details for Latent Class Analysis (LCA) 24](#_Toc172057000)

[SI.2.1 Survey questions on risk mitigation behavior at baseline 24](#_Toc172057001)

[SI.2.2 Distribution of reported risk mitigation measures at baseline 26](#_Toc172057002)

[SI.2.3 Detailed methodologies for LCA 26](#_Toc172057003)

[SI.2.4 Radar plots for LCA solutions 28](#_Toc172057004)

[SI.2.5 Model fit and diagnostics 29](#_Toc172057005)

[SI.2.6 Distribution of behavioral-related survey responses across the final LCA categories 30](#_Toc172057006)

[SI.3 Schematic for exposure classification 32](#_Toc172057007)

[SI.4 Assessing correlations between key covariates 33](#_Toc172057008)

[SI.5 Location-specific contact rates over survey round by time-invariant covariates 35](#_Toc172057009)

[SI.6 Contact rates over survey round by time-varying covariates 38](#_Toc172057010)

[SI.7 Effect of vaccination on changes in location-specific contact 43](#_Toc172057011)

[SI.8 Effect of vaccination on changes in all contacts without concern for new variants 49](#_Toc172057012)

[SI.9 Sensitivity analysis on contact outlier cutoff 51](#_Toc172057013)

[SI.10 Additional Next Generation Matrix analysis on impact on transmission 53](#_Toc172057014)

# SI.1 Comparing distribution of covariates among initially enrolled study population and those completing follow-up

| **Variable** | **Value** | **Total completing follow-up (%) (N=2403)** | **Total initially enrolled (N=4654)** |  |
| --- | --- | --- | --- | --- |
|  |  |  |  |  |
|  |  |  |  |  |
| **Age group** | 18-24 | 111 (5%) | 308(7%) |  |
|  | 25-34 | 345 (14%) | 705(15%) |  |
|  | 35-44 | 407 (17%) | 777(17%) |  |
|  | 45-54 | 417 (17%) | 765(16%) |  |
|  | 55-64 | 502 (21%) | 926(20%) |  |
|  | 65+ | 621 (26%) | 1173(25%) |  |
| **Gender** | Female | 1496 (62%) | 2727(59%) |  |
|  | Male | 907 (38%) | 1927(41%) |  |
| **Race/ethnicity** | Hispa nic | 276 (11%) | 607(13%) |  |
|  | Non-Hispanic, White | 1657 (69%) | 3063(66%) |  |
|  | Non-Hispanic, Black | 302 (13%) | 683(15%) |  |
|  | Non-Hispanic, Asian | 126 (5%) | 221(5%) |  |
|  | Non-Hispanic, Other | 42 (2%) | 80(2%) |  |
| **Household size** | 1 | 638 (27%) | 1204(26%) |  |
|  | 2-4 | 1619 (67%) | 3093(66%) |  |
|  | 5+ | 146 (6%) | 357(8%) |  |
| **Self-reported political affiliation** | Democratic | 996 (41%) | 1201(26%) |  |
|  | Republican | 378 (16%) | 464(10%) |  |
|  | Independent | 445 (19%) | 529(11%) |  |
|  | Unknown | 584 (24%) | 2460(53%) |  |
| **Employment status** | Emp,in home | 472 (20%) | 857(18%) |  |
|  | Emp,out home | 950 (40%) | 1854(40%) |  |
|  | Unemp | 891 (37%) | 1734(37%) |  |
|  | Unknown | 90 (4%) | 209(4%) |  |
| **Household income** | 0-$24,999 | 250 (10%) | 608(13%) |  |
|  | $25,000-$74,999 | 756 (31%) | 1470(32%) |  |
|  | $75,000-$149,999 | 695 (29%) | 1222(26%) |  |
|  | Greater than $150,000 | 384 (16%) | 717(15%) |  |
|  | Unknown | 318 (13%) | 637(14%) |  |
| **Comorbidities** | No | 1151 (48%) | 2237(48%) |  |
|  | Yes | 1252 (52%) | 2417(52%) |  |

Table S1. Comparison of distribution of covariates among study participants initially enrolled and study participants who completed all four rounds of follow-up that were included in the study.

# SI.2 Additional details for Latent Class Analysis (LCA)

## SI.2.1 Survey questions on risk mitigation behavior at baseline

Table S2. Survey questions and values on risk mitigation behavior that were considered in as indicator variables for the Latent Class Analysis used to classify participants into different levels of inherent risk tolerance at baseline

| Variable | Question | Values |
| --- | --- | --- |
| Social distancing | How often are you trying to keep at least 6 feet between you and other people you don't live with to avoid spreading illness? | 1 = Never 2 = Rarely 3 = Sometimes 4 = Often 5 = Always |
| Essential travel | In the last month, how often have you gone out to grocery stores, pharmacies, or visiting other essential service providers? | 1 = Daily 2 = Several times a week 3 = Once a week 4 = Once every two-three weeks 5 = Monthly or less often 6 = Never |
| Nonessential travel | In the last month, how often have you gone out to bars, dining at restaurants, exercising at gyms or other non-essential venues? | 1 = Daily 2 = Several times a week 3 = Once a week 4 = Once every two-three weeks 5 = Monthly or less often 6 = Never |
| Face mask | When you go out, do you wear a face mask? | 1 = Never (0%) 2 = Rarely (1 - 30%) 3 = Sometimes (31 - 69%) 4 = Often (70 - 99%)  5 = Always (100%) |
| Public transport | In the last month, how often have you used public transportation (bus/train) or car service (taxi/Uber/Lyft/other rideshare)? | 0 = 0 times 1 = 1 - 2 times 2 = 3 - 5 times 3 = 6 - 10 times 4 = More than 10 times |
| Intention to vaccinate | How likely are you to get vaccinated for coronavirus once a vaccination is available to the public? | 1 = Very unlikely 2 = Somewhat unlikely 3 = Somewhat likely 4 = Very likely 5 = Unsure |
| Handwashing with soap | Estimate how many times you washed your hands with soap and water yesterday | Quantiles for analysis |
| Hand sanitizing | Estimate how many times you used hand sanitizer on your hands yesterday | Tertiles for analysis |

## SI.2.2 Distribution of reported risk mitigation measures at baseline


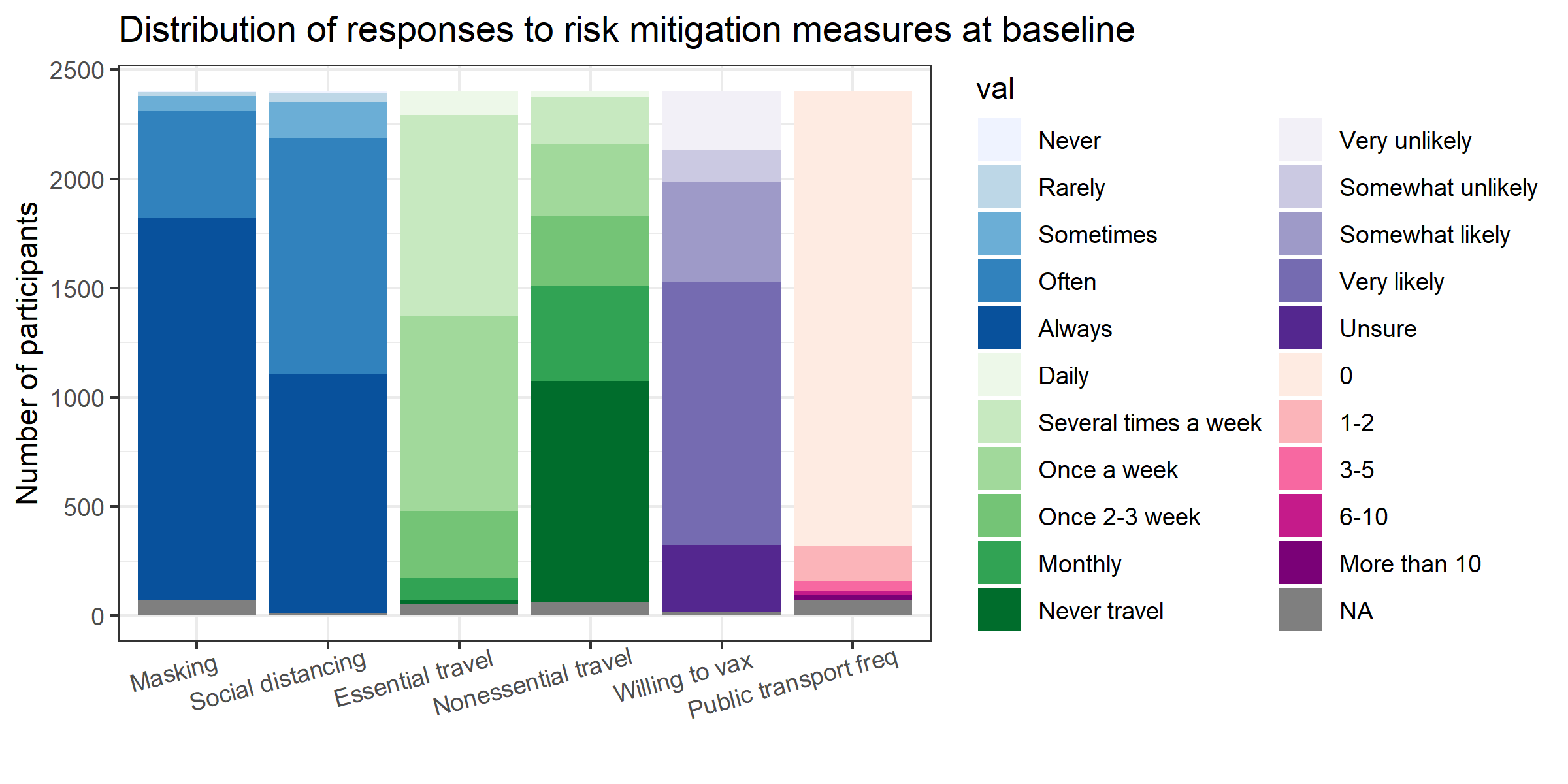
Figure S1. Distribution of indicator variables related to behavior and risk mitigation for COVID-19 prevention at baseline. Categorical responses vary based on the variable but in general, darker colors indicate more precaution and risk mitigation

## SI.2.3 Detailed methodologies for LCA

We conducted latent class analysis (LCA) on level of adoption of risk mitigation behaviors reported at baseline to classify participants as having different intrinsic levels of risk tolerance. Participants were further asked to report on the level of adoption of recommended COVID-related risk mitigation strategies such as frequency of mask-wearing, social distancing, essential and non-essential travel and handwashing with soap. LCA assumes the presence of an underlying, unobserved latent variable that can explain patterns among observed indicator variables and identifies latent typologies of similar response patterns within indicator variables , classifying participants into unobserved groups of similar patterns of COVID-19 risk mitigation behaviors. Here, we assume that the level of cautionary behavior during the acute phase of the pandemic is closely correlated with an inherent level of risk tolerance. Table 1 shows the survey questions related to risk mitigation available for the LCA analysis at baseline.

We use the R software package “poLCA”^32^ and considered several sets of indicator variables. To select for the number of classes, we considered statistical criteria of model fit and model diagnostics. For model fit, we primarily considered the Bayesian information criteria (BIC) which penalizes the log-likelihood by a function of the number of parameters estimated. We further considered the mean posterior probability for classification (target >80%); the entropy (target >0.8 but minimum >0.6) as diagnostic criteria and ensured that no class sizes are smaller than 50^33^. To decide on the set of indicator variables and number of classes, we aimed to select the best-fitting model that met diagnostic criteria that allowed for more classes for more distinguishing power. Individuals are assigned to latent classes based on the probabilities of belonging to each class based on the model of choice.

| Model | Indicator variables |
| --- | --- |
| Full model | All indicator variables |
| Model 1 | Social distancing + Essential travel + Nonessential travel +Face mask + Intention to vaccinate + Handwashing with soap + Hand sanitizing |
| Model 2 | Social distancing + Essential travel + Nonessential travel +Face mask + Intention to vaccinate |
| Model 3 | Social distancing + Essential travel + Nonessential travel +Face |

Table S3. Different sets of indicator variables considered for the Latent Class Analysis

## SI.2.4 Radar plots for LCA solutions


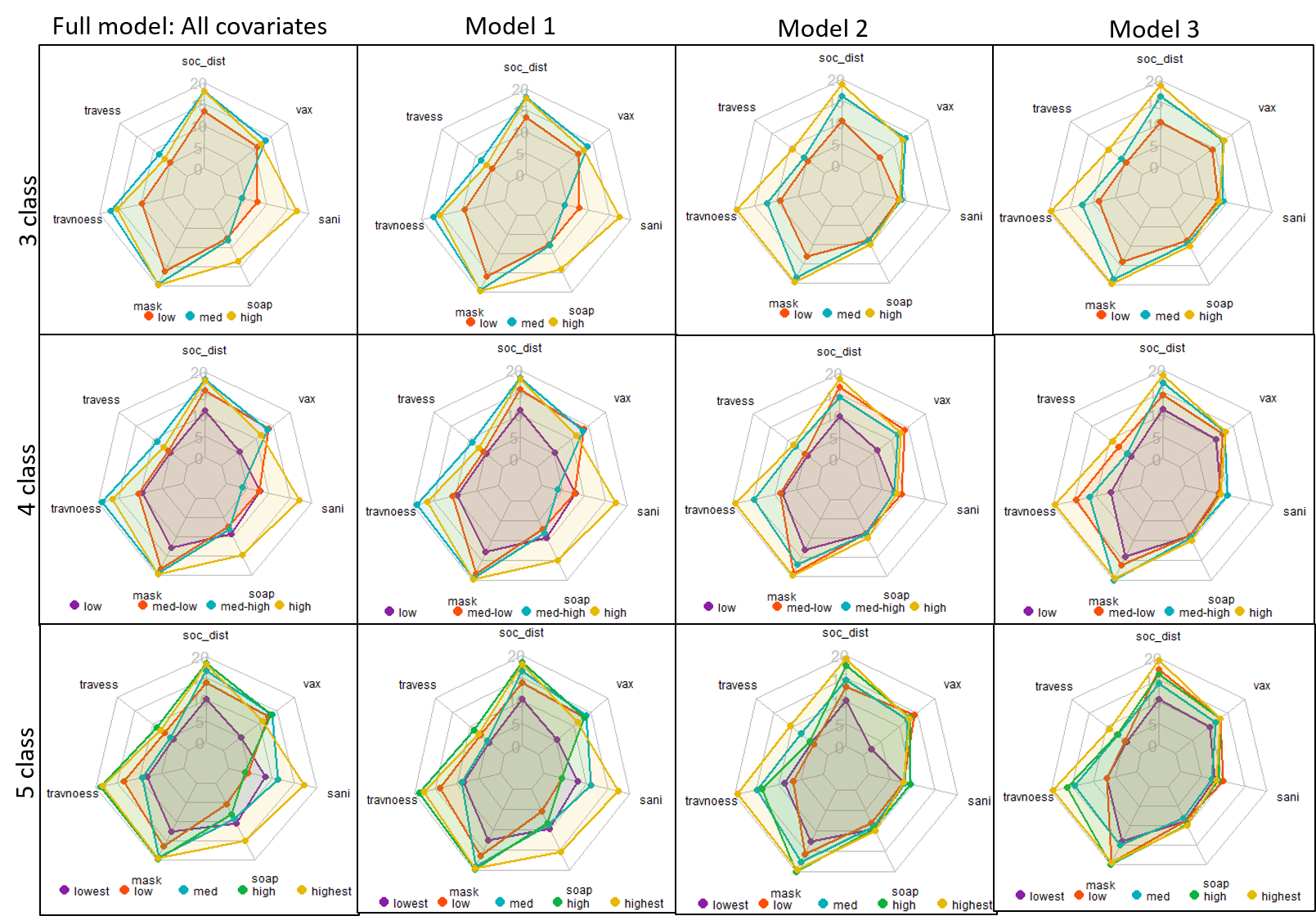


Figure S2. Radar plots visualizing the average profiles of each class for the three-class solution classified under four different groupings of indicator variables. Latent class models including frequency of handwashing with soap and hand sanitization added an additional dimension to the classification scheme (non-concentric area plots).

## SI.2.5 Model fit and diagnostics

|  |  | **1 class** | **2 class** | **3 class** | **4 class** | **5 class** | **6 class** |
| --- | --- | --- | --- | --- | --- | --- | --- |
| **BIC** | **Full model** | 40867 | 40352 | 40352 | 40418 | 40575 | 40781 |
|  | **Model 1** | 38742 | 38219 | 38197 | 38239 | 38361 | 38531 |
|  | **Model 2** | 27824 | 27269 | 27266 | 27358 | 27491 | 27640 |
|  | **Model 3** | 21379 | 20835 | 20876 | 20940 | 21064 | 21166 |
| **AIC** | **Full model** | 40681 | 39976 | 39785 | 39661 | 39627 | 39641 |
|  | **Model 1** | 38580 | 37889 | 37700 | 37573 | 37528 | 37530 |
|  | **Model 2** | 27697 | 27009 | 26873 | 26832 | 26831 | 26847 |
|  | **Model 3** | 21275 | 20621 | 20552 | 20506 | 20520 | 20512 |
| **Smalles class (N,%)** | **Full model** | 2403 | 808 (34%) | 645 (27%) | 219 (9%) | 175 (7%) | 102 (4%) |
|  | **Model 1** | 2403 | 756 (31%) | 567 (24%) | 223 (9%) | 187 (8%) | 167 (7%) |
|  | **Model 2** | 2403 | 763 (32%) | 239 (10%) | 197 (8%) | 120 (5%) | 105 (4%) |
|  | **Model 3** | 2403 | 879 (37%) | 326 (14%) | 291 (12%) | 239 (10%) | 50 (2%) |
| **Mean posterior probability** | **Full model** | 1 | 0.87 | 0.82 | 0.8 | 0.73 | 0.69 |
|  | **Model 1** | 1 | 0.88 | 0.82 | 0.8 | 0.74 | 0.68 |
|  | **Model 2** | 1 | 0.88 | 0.8 | 0.77 | 0.67 | 0.65 |
|  | **Model 3** | 1 | 0.86 | 0.78 | 0.71 | 0.67 | 0.72 |
| **Entropy** | **Full model** | NA | 0.55 | 0.6 | 0.6 | 0.56 | 0.53 |
|  | **Model 1** | NA | 0.56 | 0.58 | 0.6 | 0.58 | 0.55 |
|  | **Model 2** | NA | 0.55 | 0.59 | 0.53 | 0.49 | 0.47 |
|  | **Model 3** | NA | 0.53 | 0.5 | 0.48 | 0.45 | 0.53 |

Table S4. Statistical criterion for model fit (BIC, AIC) and model diagnostic criteria (smallest class size, mean posterior probability and entropy) using four different groupings of indicator variables for classification and for between 1-6 classes for each grouping

## SI.2.6 Distribution of behavioral-related survey responses across the final LCA categories

| **Survey question** | **Value** | **4-Low risk tolerance** | **3** | **2** | **1-High risk tolerance** |
| --- | --- | --- | --- | --- | --- |
| When you go out, do you wear a face mask? | Never (0%) | 0.20% | 0% | 0% | 1.70% |
|  | Rarely (1-30%) | 0.20% | 0% | 0.10% | 7.80% |
|  | Sometimes (31-69%) | 0% | 0.60% | 1.60% | 21.30% |
|  | Often (70-99%) | 6.90% | 9.20% | 29.20% | 56.10% |
|  | Always (100%) | 90.10% | 87.70% | 66.30% | 8.30% |
|  | Unknown | 2.60% | 2.60% | 2.70% | 4.80% |
| How often are you trying to keep at least 6 feet between you and other people you don't live with to avoid spreading illness? | Never | 0.40% | 0.10% | 0% | 3.50% |
|  | Rarely | 0.80% | 0.40% | 0.10% | 14.30% |
|  | Sometimes | 1.20% | 0.50% | 7.30% | 40% |
|  | Often | 32% | 30.70% | 67.50% | 40.90% |
|  | Always | 65.20% | 68.30% | 24.50% | 0% |
|  | Unknown | 0.40% | 0% | 0.60% | 1.30% |
| In the last month, how often have you gone out to bars, dining at restaurants, exercising at gyms or other non-essential venues? | Daily | 1.20% | 0% | 1.40% | 4.30% |
|  | Several times a week | 4.20% | 0% | 15.50% | 27.80% |
|  | Once a week | 7.90% | 0.20% | 27.10% | 24.30% |
|  | Once 2-3 week | 10.70% | 1% | 25.80% | 16.10% |
|  | Monthly | 19.40% | 17.10% | 19.70% | 14.80% |
|  | Never | 54.50% | 79.20% | 8% | 7.80% |
|  | Unknown | 2.20% | 2.40% | 2.50% | 4.80% |
| In the last month, how often have you gone out to grocery stores, pharmacies, or visiting other essential service providers? | Daily | 5.50% | 0% | 5.80% | 14.80% |
|  | Several times a week | 40.70% | 22.20% | 48.80% | 52.20% |
|  | Once a week | 32.80% | 41.90% | 39.50% | 20.40% |
|  | Once 2-3 week | 13.40% | 23.20% | 3.80% | 6.10% |
|  | Monthly | 5.70% | 8.70% | 0% | 1.30% |
|  | Never travel | 0.20% | 2.20% | 0.10% | 0% |
|  | Unknown | 1.60% | 1.70% | 2.10% | 5.20% |
| In the last month, how often have you used public transportation (bus/train) or car service (taxi/Uber/Lyft/other rideshare)? | 0 times | 88.70% | 92.50% | 82.30% | 78.70% |
|  | 1-2 times | 4.70% | 3.20% | 10% | 10.90% |
|  | 3-5 times | 0.80% | 1.30% | 2.60% | 3% |
|  | 6-10 times | 0.40% | 0.20% | 1.30% | 1.30% |
|  | More than 10 | 3% | 0.10% | 1.10% | 0.90% |
|  | Unknown | 2.40% | 2.60% | 2.70% | 5.20% |
| How likely are you to get vaccinated for coronavirus once a vaccination is available to the public? | Very unlikely | 18.60% | 7% | 3.50% | 39.10% |
|  | Somewhat unlikely | 7.90% | 4.20% | 2% | 23.50% |
|  | Somewhat likely | 18.80% | 16% | 21.80% | 20.40% |
|  | Very likely | 35.40% | 59.90% | 60.10% | 11.70% |
|  | Unsure | 18.80% | 12.30% | 12% | 4.30% |
|  | Unknown | 0.60% | 0.60% | 0.60% | 0.90% |
| Estimate how many times you used hand sanitizer on your hands yesterday | 0 times | 0% | 35.70% | 20.50% | 28.70% |
|  | 1-3 times | 1% | 42.70% | 41.60% | 24.30% |
|  | 4-5 times | 36% | 2.30% | 8.80% | 12.20% |
|  | 6 or more times | 54.90% | 0% | 10.40% | 15.70% |
|  | Unknown | 8.10% | 19.30% | 18.70% | 19.10% |
| Estimate how many times you washed your hands with soap and water yesterday | 0-4 times | 1.80% | 24.90% | 30.30% | 23.90% |
|  | 5-6 times | 14.20% | 23% | 25.80% | 20% |
|  | 7-10 times | 39.90% | 22.70% | 17.70% | 23% |
|  | 11 or more times | 36.80% | 10.90% | 8.10% | 15.70% |
|  | Unknown | 7.30% | 18.50% | 18.10% | 17.40% |

Table S5. Distribution of behavioral-related survey responses across the final LCA categories

# SI.3 Schematic for exposure classification


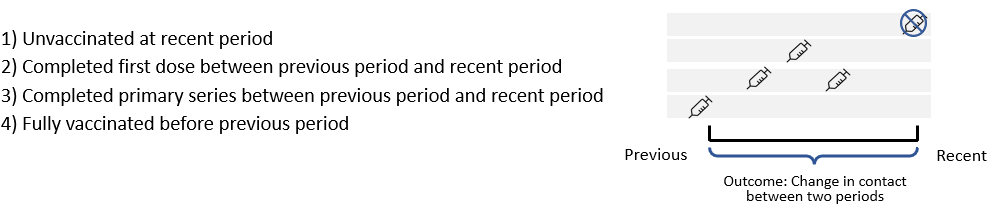


Figure S3. Schematic for exposure classification

# SI.4 Assessing correlations between key covariates

Table S6. Correlation between risk tolerance at baseline categorized through Latent Class Analysis and changes in concern for new variants in the pandemic.

|  | **Risk tolerance at baseline** | **Total** | **Concern**  **Increased greatly** | **Concern**  **Increased slightly** | **No change in concern** | **Concern Decreased slightly** | **Concern Decreased greatly** | **Unknown** | **Spearman's Rank Correlation Coefficient** |
| --- | --- | --- | --- | --- | --- | --- | --- | --- | --- |
|  |  |  | **n (%)** | **n (%)** | **n (%)** | **n (%)** | **n (%)** | **n (%)** |  |
| **Round 2** | High | 208 | 3(1%) | 53(25%) | 128(62%) | 1(0%) | 4(2%) | 19(9%) | -0.1625 |
|  | Med-high | 841 | 52(6%) | 432(51%) | 313(37%) | 3(0%) | 4(0%) | 37(4%) |  |
|  | Med-low | 856 | 96(11%) | 480(56%) | 240(28%) | 7(1%) | 1(0%) | 32(4%) |  |
|  | Low | 498 | 68(14%) | 226(45%) | 165(33%) | 3(1%) | 2(0%) | 34(7%) |  |
| **Round 3** | High | 208 | 14(7%) | 70(34%) | 115(55%) | 3(1%) | 5(2%) | 1(0%) | -0.157 |
|  | Med-high | 841 | 122(15%) | 493(59%) | 196(23%) | 15(2%) | 14(2%) | 1(0%) |  |
|  | Med-low | 856 | 201(23%) | 489(57%) | 142(17%) | 12(1%) | 11(1%) | 1(0%) |  |
|  | Low | 498 | 116(23%) | 245(49%) | 122(24%) | 7(1%) | 7(1%) | 1(0%) |  |
| **Round 4** | High | 208 | 1(0%) | 16(8%) | 138(66%) | 19(9%) | 34(16%) | 0(0%) | -0.1691 |
|  | Med-high | 841 | 19(2%) | 204(24%) | 446(53%) | 100(12%) | 71(8%) | 1(0%) |  |
|  | Med-low | 856 | 49(6%) | 287(34%) | 403(47%) | 71(8%) | 43(5%) | 3(0%) |  |
|  | Low | 498 | 31(6%) | 147(30%) | 247(50%) | 41(8%) | 31(6%) | 1(0%) |  |

Table S7. Correlation between risk tolerance at baseline categorized through Latent Class Analysis and vaccination status over round.

|  | **Risk tolerance at baseline** | **Total** | **Remain unvaccinated** | **First dose new** | **Newly completed series** | **Already fully vaccinated** | **Spearman's Rank Correlation Coefficient** |
| --- | --- | --- | --- | --- | --- | --- | --- |
|  |  |  | **n (%)** | **n (%)** | **n (%)** | **n (%)** |  |
| **Round 2** | High | 208 | 159(76%) | 21(10%) | 28(13%) | 0(0%) | 0.0865 |
|  | Med-high | 841 | 398(47%) | 173(21%) | 270(32%) | 0(0%) |  |
|  | Med-low | 856 | 369(43%) | 210(25%) | 277(32%) | 0(0%) |  |
|  | Low | 498 | 247(50%) | 80(16%) | 171(34%) | 0(0%) |  |
| **Round 3** | High | 208 | 79(38%) | 15(7%) | 86(41%) | 28(13%) | 0.09582 |
|  | Med-high | 841 | 58(7%) | 61(7%) | 452(54%) | 270(32%) |  |
|  | Med-low | 856 | 45(5%) | 41(5%) | 493(58%) | 277(32%) |  |
|  | Low | 498 | 72(14%) | 32(6%) | 223(45%) | 171(34%) |  |
| **Round 4** | High | 208 | 53(25%) | 13(6%) | 28(13%) | 114(55%) | 0.087 |
|  | Med-high | 841 | 31(4%) | 18(2%) | 69(8%) | 723(86%) |  |
|  | Med-low | 856 | 26(3%) | 11(1%) | 49(6%) | 770(90%) |  |
|  | Low | 498 | 38(8%) | 8(2%) | 58(12%) | 394(79%) |  |

Table S8 Correlation between concern for new variants in the pandemic and vaccination status over round.

|  | **Concern for pandemic** | **Total** | **Remain unvaccinated** | **First dose new** | **Newly completed series** | **Already fully vaccinated** | **Spearman's Rank Correlation Coefficient** |
| --- | --- | --- | --- | --- | --- | --- | --- |
|  |  |  | **n (%)** | **n (%)** | **n (%)** | **n (%)** |  |
| **Round 2** | Increased greatly | 219 | 123 (56%) | 48 (22%) | 48 (22%) | 0 (0%) | -0.00675 |
|  | Increased slightly | 1191 | 527 (44%) | 246 (21%) | 418 (35%) | 0 (0%) |  |
|  | No change | 846 | 415 (49%) | 173 (20%) | 258 (30%) | 0 (0%) |  |
|  | Decreased slightly | 14 | 6 (43%) | 2 (14%) | 6 (43%) | 0 (0%) |  |
|  | Decreased greatly | 11 | 10 (91%) | 1 (9%) | 0 (0%) | 0 (0%) |  |
| **Round 3** | Increased greatly | 453 | 25 (6%) | 24 (5%) | 255 (56%) | 149 (33%) | -0.1587 |
|  | Increased slightly | 1297 | 81 (6%) | 69 (5%) | 705 (54%) | 442 (34%) |  |
|  | No change | 575 | 129 (22%) | 54 (9%) | 263 (46%) | 129 (22%) |  |
|  | Decreased slightly | 37 | 10 (27%) | 1 (3%) | 15 (41%) | 11 (30%) |  |
|  | Decreased greatly | 37 | 8 (22%) | 1 (3%) | 14 (38%) | 14 (38%) |  |
| **Round 4** | Increased greatly | 100 | 5 (5%) | 1 (1%) | 14 (14%) | 80 (80%) | -0.108 |
|  | Increased slightly | 654 | 12 (2%) | 6 (1%) | 36 (6%) | 600 (92%) |  |
|  | No change | 1234 | 96 (8%) | 30 (2%) | 125 (10%) | 983 (80%) |  |
|  | Decreased slightly | 231 | 11 (5%) | 6 (3%) | 12 (5%) | 202 (87%) |  |
|  | Decreased greatly | 179 | 24 (13%) | 7 (4%) | 17 (9%) | 131 (73%) |  |

# SI.5 Location-specific contact rates over survey round by time-invariant covariates

Table S9. Mean contact rates reported at work (95% CI) over each survey round stratified by sociodemographic and other individual covariates (ex. political affiliation, comorbidities and risk tolerance based on latent class analysis of responses to survey questions related to risk mitigation)

| **Variable** | **Value** | **Total (%) (N=2403)** | **Mean contact (95% CI)** | | | |
| --- | --- | --- | --- | --- | --- | --- |
|  |  |  | **Round 1** | **Round 2** | **Round 3** | **Round 4** |
|  |  |  | **(Aug-Dec, 2020)** | **(Mar-Apr, 2021)** | **(July-Aug, 2021)** | **(Mar-April, 2022)** |
| **Overall** | | 2403 (100%) | 4 (3.4-4.5) | 4.8 (4.1-5.4) | 5.3 (4.6-6) | 7.5 (6.7-8.4) |
| **Age group** | 18-24 | 111 (5%) | 6 (2.9-9.1) | 6.1 (2.9-9.2) | 9.2 (4.8-13.6) | 10.9 (6.5-15.4) |
|  | 25-34 | 345 (14%) | 6.4 (4.6-8.1) | 8.6 (6.3-10.9) | 10.5 (7.8-13.2) | 11.2 (8.8-13.7) |
|  | 35-44 | 407 (17%) | 5 (3.7-6.4) | 5.3 (3.8-6.8) | 6.3 (4.5-8) | 11 (8.4-13.6) |
|  | 45-54 | 417 (17%) | 5.7 (4.1-7.2) | 5.9 (4.3-7.6) | 5.9 (4.3-7.6) | 10.6 (8-13.2) |
|  | 55-64 | 502 (21%) | 3.8 (2.7-4.9) | 5.3 (3.6-6.9) | 4.4 (3.2-5.6) | 6.6 (4.8-8.5) |
|  | 65+ | 621 (26%) | 0.6 (0.2-0.9) | 0.9 (0.5-1.3) | 1.3 (0.8-1.9) | 1.2 (0.7-1.7) |
| **Gender** | Female | 1496 (62%) | 4 (3.3-4.6) | 5.3 (4.5-6.2) | 5.3 (4.5-6.2) | 7.8 (6.7-8.9) |
|  | Male | 907 (38%) | 4 (3.1-4.8) | 3.9 (3-4.8) | 5.2 (4.1-6.2) | 7.1 (5.7-8.4) |
| **Race/ethnicity** | 1_Hisp | 276 (11%) | 3.7 (2.5-5) | 5.1 (3.3-6.9) | 6.4 (4.2-8.6) | 9.8 (6.8-12.9) |
|  | 2_NH-White | 1657 (69%) | 3.9 (3.3-4.6) | 4.5 (3.8-5.2) | 5.2 (4.4-6) | 7.4 (6.3-8.4) |
|  | 3_NH-Black | 302 (13%) | 4.4 (2.7-6.1) | 6.5 (4.1-8.8) | 4.7 (2.9-6.4) | 7.6 (5-10.2) |
|  | 4_NH-Asian | 126 (5%) | 4 (2-6) | 4.2 (1.5-6.8) | 5.7 (2.4-9.1) | 5.6 (3.2-8) |
|  | 5_NH-Other | 42 (2%) | 3.1 (0-6.2) | 4.1 (-1.5-9.7) | 3.8 (-1.8-9.4) | 3.3 (0.1-6.4) |
| **Household size** | 1 | 638 (27%) | 3.5 (2.5-4.5) | 4.2 (3-5.3) | 5.2 (3.9-6.5) | 6.8 (5.2-8.4) |
|  | 2-4 | 1619 (67%) | 4.1 (3.5-4.7) | 4.7 (3.9-5.4) | 5.2 (4.4-6) | 7.4 (6.3-8.4) |
|  | 5+ | 146 (6%) | 4.4 (2-6.8) | 8.6 (4.6-12.6) | 6.6 (3.3-9.9) | 12.4 (7.2-17.5) |
| **Political affiliation** | Dem | 996 (41%) | 3.4 (2.6-4.1) | 4 (3.1-4.8) | 4.5 (3.5-5.5) | 7.4 (6-8.8) |
|  | Rep | 378 (16%) | 3.4 (2.2-4.5) | 4 (2.7-5.3) | 4.7 (3.3-6.1) | 6.5 (4.6-8.4) |
|  | Ind | 445 (19%) | 3.5 (2.4-4.6) | 4.8 (3.2-6.4) | 5.7 (4.1-7.4) | 8.2 (6.1-10.2) |
|  | Unknown | 584 (24%) | 5.7 (4.4-7) | 6.6 (5-8.2) | 6.6 (5-8.2) | 7.9 (6.2-9.7) |
| **Employment status** | Emp,in home | 472 (20%) | 0.4 (0-0.8) | 0.9 (0.4-1.4) | 2.8 (1.5-4) | 6.2 (4.3-8.1) |
|  | Emp,out home | 950 (40%) | 9.3 (8.1-10.5) | 10.4 (8.9-11.8) | 10.7 (9.3-12.1) | 13.4 (11.7-15.2) |
|  | Unemp | 891 (37%) | 0.2 (0-0.4) | 0.6 (0.3-0.9) | 0.9 (0.4-1.4) | 1.5 (1-2.1) |
|  | Unknown | 90 (4%) | 4 (2.2-5.7) | 7.6 (3.3-12) | 5.2 (1.6-8.7) | 11.5 (6.1-16.9) |
| **Comorbidities** | No | 1151 (48%) | 4.4 (3.6-5.2) | 5.2 (4.2-6.1) | 6 (4.9-7) | 8.2 (7-9.5) |
|  | Yes | 1252 (52%) | 3.6 (2.9-4.3) | 4.4 (3.6-5.2) | 4.6 (3.8-5.5) | 6.8 (5.7-8) |
| **Risk tolerance (from Latent Class Analysis)** | High | 208 (9%) | 6.7 (4.4-9) | 9.4 (6.7-12.1) | 10.3 (7-13.6) | 11.7 (8.3-15.2) |
|  | Med-high | 841 (35%) | 3.8 (3-4.7) | 4.5 (3.5-5.5) | 4.8 (3.8-5.7) | 7.5 (6.2-8.9) |
|  | Med-low | 856 (36%) | 1.4 (0.9-1.9) | 2.6 (1.8-3.5) | 2.8 (2-3.6) | 5.1 (3.9-6.4) |
|  | Low | 498 (21%) | 7.5 (5.8-9.1) | 7 (5.3-8.8) | 8.3 (6.4-10.2) | 9.8 (7.6-12.1) |

Table S10 Mean contact rates reported at other locations (95% CI) over each survey round stratified by sociodemographic and other individual covariates (ex. political affiliation, comorbidities and risk tolerance based on latent class analysis of responses to a set of survey questions related to risk mitigation behavior)

| **Variable** | **Value** | **Total (%) (N=2403)** | **Mean contact (95% CI)** | | | |
| --- | --- | --- | --- | --- | --- | --- |
|  |  |  | **Round 1** | **Round 2** | **Round 3** | **Round 4** |
|  |  |  | **(Aug-Dec, 2020)** | **(Mar-Apr, 2021)** | **(July-Aug, 2021)** | **(Mar-April, 2022)** |
| **Overall** | | 2403 (100%) | 2.4 (2.2-2.6) | 2.7 (2.4-3) | 4.1 (3.7-4.4) | 4.4 (4-4.9) |
| **Age group** | 18-24 | 111 (5%) | 3.5 (2.2-4.9) | 3.4 (2-4.9) | 3.9 (2.2-5.5) | 2.8 (1.7-4) |
|  | 25-34 | 345 (14%) | 2.3 (1.8-2.9) | 2.3 (1.7-2.9) | 3.6 (2.6-4.6) | 3 (2.1-3.9) |
|  | 35-44 | 407 (17%) | 2.7 (2.1-3.3) | 3.1 (2.4-3.8) | 4 (3.1-4.8) | 4.5 (3.4-5.7) |
|  | 45-54 | 417 (17%) | 2.5 (2-3) | 2.5 (1.9-3.1) | 4 (3-5) | 3.9 (3-4.8) |
|  | 55-64 | 502 (21%) | 2.5 (2-2.9) | 2.5 (1.9-3.1) | 4.3 (3.4-5.2) | 4.5 (3.5-5.4) |
|  | 65+ | 621 (26%) | 2 (1.6-2.3) | 2.9 (2.3-3.4) | 4.2 (3.6-4.9) | 5.7 (4.8-6.7) |
| **Gender** | Female | 1496 (62%) | 2.4 (2.1-2.7) | 2.7 (2.3-3) | 4 (3.5-4.4) | 4.3 (3.8-4.9) |
|  | Male | 907 (38%) | 2.4 (2.1-2.7) | 2.7 (2.3-3.2) | 4.2 (3.6-4.8) | 4.6 (3.9-5.3) |
| **Race/ethnicity** | 1_Hisp | 276 (11%) | 2.1 (1.6-2.7) | 2.4 (1.7-3.2) | 3.3 (2.4-4.3) | 3.3 (2.3-4.4) |
|  | 2_NH-White | 1657 (69%) | 2.4 (2.1-2.6) | 2.6 (2.3-2.9) | 4.3 (3.8-4.7) | 4.8 (4.2-5.3) |
|  | 3_NH-Black | 302 (13%) | 2.8 (2.1-3.6) | 3.1 (2.1-4.1) | 3.7 (2.6-4.8) | 4.4 (3.1-5.7) |
|  | 4_NH-Asian | 126 (5%) | 2.7 (1.5-3.8) | 2.9 (1.6-4.2) | 3 (2-3.9) | 2.9 (1.7-4.1) |
|  | 5_NH-Other | 42 (2%) | 2.5 (1-4) | 4.1 (1.1-7.2) | 6.3 (1.9-10.7) | 3.5 (1.6-5.3) |
| **Household size** | 1 | 638 (27%) | 2.4 (2-2.8) | 2.9 (2.4-3.5) | 3.8 (3.2-4.5) | 3.9 (3.2-4.7) |
|  | 2-4 | 1619 (67%) | 2.4 (2.1-2.7) | 2.6 (2.2-2.9) | 4.2 (3.7-4.7) | 4.6 (4-5.1) |
|  | 5+ | 146 (6%) | 2.3 (1.5-3.1) | 3.4 (2.1-4.7) | 3.5 (2.2-4.9) | 5.1 (2.9-7.3) |
| **Political affiliation** | Dem | 996 (41%) | 2.3 (1.9-2.6) | 2.5 (2-2.9) | 3.5 (3-4) | 3.5 (3-4) |
|  | Rep | 378 (16%) | 2.6 (2-3.2) | 3.1 (2.4-3.8) | 5.5 (4.4-6.6) | 6 (4.8-7.2) |
|  | Ind | 445 (19%) | 2.5 (2-3) | 2.5 (2-3.1) | 4 (3.2-4.9) | 4.6 (3.6-5.6) |
|  | Unknown | 584 (24%) | 2.5 (2-2.9) | 3 (2.4-3.6) | 4 (3.2-4.8) | 4.9 (3.8-5.9) |
| **Employment status** | Emp,in home | 472 (20%) | 2.4 (2-2.9) | 2.3 (1.8-2.9) | 3.3 (2.6-4.1) | 3.8 (2.9-4.7) |
|  | Emp,out home | 950 (40%) | 2.6 (2.2-3) | 2.6 (2.2-3) | 4.1 (3.4-4.7) | 4.2 (3.6-4.9) |
|  | Unemp | 891 (37%) | 2.1 (1.8-2.4) | 2.9 (2.5-3.4) | 4.4 (3.8-5) | 4.9 (4.2-5.5) |
|  | Unknown | 90 (4%) | 2.8 (1.6-4.1) | 3.4 (1.7-5.2) | 4.5 (2.2-6.8) | 5.4 (2.4-8.4) |
| **Comorbidities** | No | 1151 (48%) | 2.5 (2.2-2.8) | 2.7 (2.3-3) | 4 (3.4-4.5) | 4.4 (3.7-5) |
|  | Yes | 1252 (52%) | 2.3 (2-2.6) | 2.7 (2.4-3.1) | 4.1 (3.6-4.7) | 4.5 (3.9-5.1) |
| **Risk tolerance (from Latent Class Analysis)** | High | 208 (9%) | 4.5 (3.4-5.5) | 5.5 (4.1-7) | 7.1 (5.2-9) | 8.1 (5.9-10.4) |
|  | Med-high | 841 (35%) | 3.2 (2.8-3.7) | 2.9 (2.5-3.3) | 4.2 (3.7-4.8) | 4.7 (4-5.4) |
|  | Med-low | 856 (36%) | 1.1 (0.9-1.3) | 1.9 (1.5-2.3) | 3.4 (2.8-4) | 3.4 (2.8-4) |
|  | Low | 498 (21%) | 2.4 (1.9-2.9) | 2.6 (2-3.2) | 3.7 (2.8-4.5) | 4.2 (3.2-5.1) |

# SI.6 Contact rates over survey round by time-varying covariates

|  |  | | **Round 1** | | | **Round 2** | | | **Round 3** | | | **Round 4** | |  |
| --- | --- | --- | --- | --- | --- | --- | --- | --- | --- | --- | --- | --- | --- | --- |
| **Variable** | **Value** | **Total (%)** | | **Mean contact (95% CI)** | **Total (%)** | | **Mean contact (95% CI)** | **Total (%)** | | **Mean contact (95% CI)** | **Total (%)** | | **Mean contact (95% CI)** | |
| **Overall** |  | 2403 (100%) | | 8.4 (7.8-9) | 2403 (100%) | | 9.8 (9.1-10.6) | 2403 (100%) | | 11.7 (10.8-12.5) | 2403 (100%) | | 14.7 (13.7-15.8) | |
| Oxford Stringency index | High | 24 (1%) | | 10 (2-17.9) | 2 (0%) | | 26 (20.1-31.9) | - | | - | - | | - | |
|  | Med-high | 457 (19%) | | 6.4 (5.5-7.4) | 718 (30%) | | 7.6 (6.6-8.6) | - | | - | - | | - | |
|  | Med-low | 1647 (69%) | | 8.6 (7.8-9.3) | 555 (23%) | | 10.4 (8.7-12.1) | 13 (1%) | | 6.3 (3.2-9.4) | - | | - | |
|  | Low | 275 (11%) | | 10.4 (8.1-12.7) | 1128 (47%) | | 10.9 (9.7-12.1) | 2390 (99%) | | 11.7 (10.9-12.5) | 2403 (100%) | | 14.7 (13.7-15.8) | |
| Self-reported level of concern for new variants | Increased greatly | - | | - | 219 (9%) | | 9.1 (6.6-11.6) | 453 (19%) | | 10.4 (8.5-12.3) | 100 (4%) | | 15.4 (9.2-21.6) | |
|  | Increased slightly | - | | - | 1191 (50%) | | 8.4 (7.5-9.3) | 1297 (54%) | | 10.8 (9.8-11.8) | 654 (27%) | | 11.7 (10.1-13.4) | |
|  | No change | - | | - | 846 (35%) | | 11.1 (9.7-12.4) | 575 (24%) | | 13.7 (11.7-15.7) | 1234 (51%) | | 15.2 (13.8-16.6) | |
|  | Decreased slightly | - | | - | 14 (1%) | | 15.4 (-0.3-31.1) | 37 (2%) | | 14.2 (8-20.5) | 231 (10%) | | 16.7 (12.7-20.7) | |
|  | Decreased greatly | - | | - | 11 (0%) | | 15 (-3.3-33.3) | 37 (2%) | | 22.8 (12-33.5) | 179 (7%) | | 20.2 (16.2-24.3) | |
|  | Unknown | 2403 (100%) | | 8.4 (7.8-9) | 122 (5%) | | 15 (9.9-20.1) | 4 (0%) | | 4 (-1.9-9.9) | 5 (0%) | | 4 (1-7) | |
| Self-reported vaccination status | None | - | | - | 1173 (49%) | | 10.8 (9.5-12) | 255 (11%) | | 15.2 (11.7-18.6) | 153 (6%) | | 15.9 (11.9-20) | |
|  | One dose | - | | - | 484 (20%) | | 9.3 (7.8-10.9) | 153 (6%) | | 11.5 (8.5-14.5) | 54 (2%) | | 13 (7.6-18.3) | |
|  | Series complete | - | | - | 746 (31%) | | 8.6 (7.6-9.5) | 1995 (83%) | | 11.2 (10.4-12.1) | 2196 (91%) | | 14.7 (13.6-15.8) | |
|  | Unknown | 2403 (100%) | | 8.4 (7.8-9) | - | | - | - | | - | - | | - | |
| County-level vaccination coverage | 0%-20% | - | | - | 2313 (96%) | | 9.7 (9-10.5) | 304 (13%) | | 12.7 (10-15.3) | - | | - | |
|  | 21%-40% | - | | - | 84 (3%) | | 11.9 (6.4-17.3) | 705 (29%) | | 12.4 (10.7-14) | 74 (3%) | | 16.1 (9.9-22.3) | |
|  | 41%-50% | - | | - | - | | - | 510 (21%) | | 11 (9.4-12.6) | 284 (12%) | | 18 (14.8-21.3) | |
|  | 51%-60% | - | | - | - | | - | 600 (25%) | | 11 (9.5-12.5) | 797 (33%) | | 15.1 (13.2-16.9) | |
|  | 61%-100% | - | | - | - | | - | 278 (12%) | | 11.3 (8.8-13.8) | 1239 (52%) | | 13.7 (12.3-15) | |
|  | Unknown | 2403 (100%) | | 8.4 (7.8-9) | 6 (0%) | | 13.5 (4.6-22.4) | 6 (0%) | | 18.2 (-3.2-39.5) | 9 (0%) | | 13.9 (-0.2-28) | |

Table S11. Mean contact rates reported at all locations (95% CI) over each survey round stratified by time-varying covariates of the Oxford Stringency index (OSI), a standardized weighted metric of level of stringency of state-level COVID-19 risk mitigation policies, self-reported individual level of concern for new variants, self-reported vaccination status and county-level vaccination coverage

Figure S4. Mean contact rates over survey round by self-reported changing concern for new variants. Across survey round, participant attitude shifted from more concern to less concern (number of respondents denoted by the size of the dot). Within each survey round, individuals who had decreased concern had higher overall contacts.


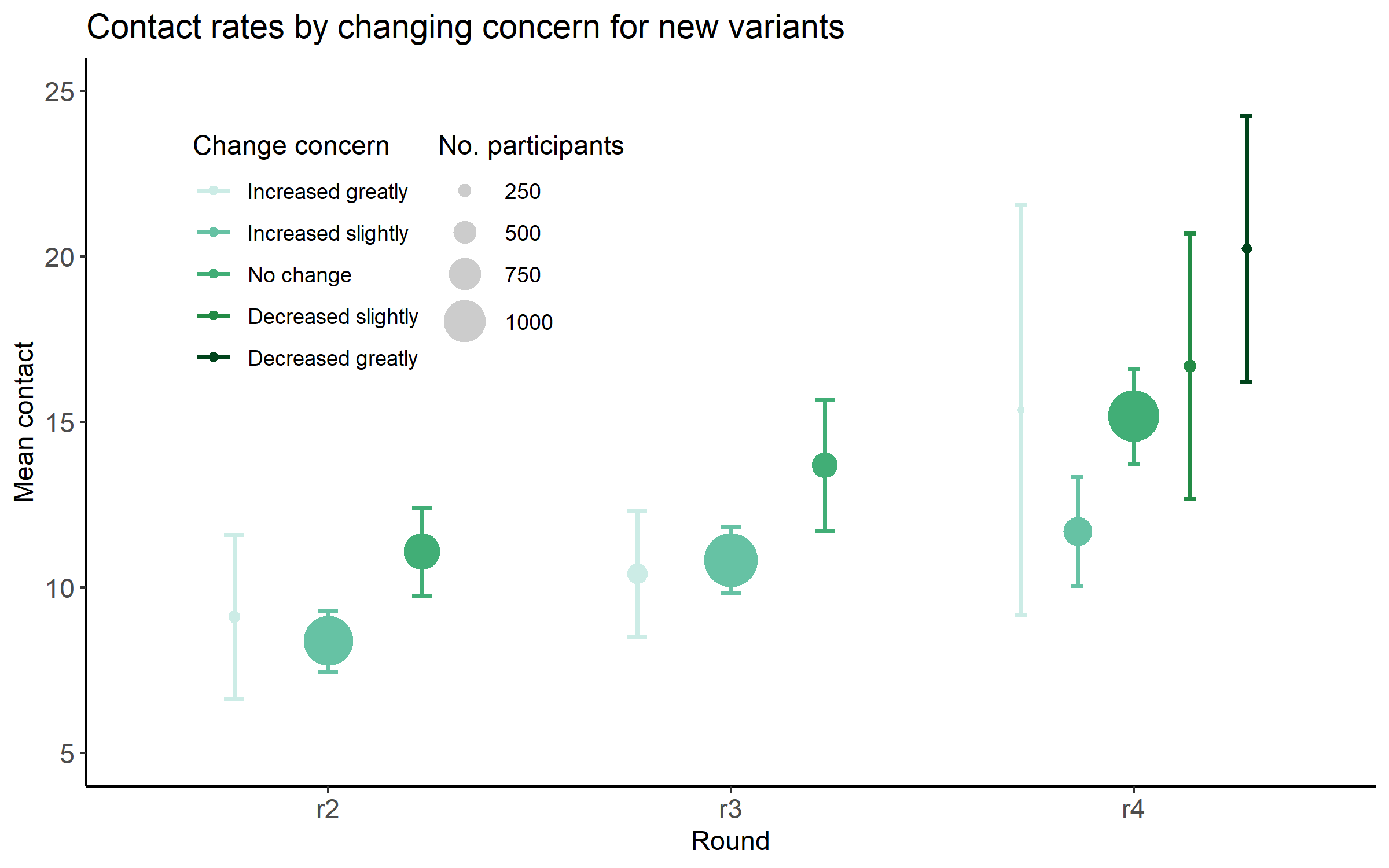


Figure S5. Mean contact rates over survey round by stringency of state-level COVID-19 policy at time of survey. Across survey round, more participants resided in states with less stringent COVID-19 policy (number of respondents denoted by the size of the dot), aligning with relaxation of risk mitigation policies during the study period (August 2020 to March 2022). In earlier survey rounds (round 1 and 2), participants who lived in states with less stringent policies had higher contact compared to those who lived in states with more stringent policies. By round 3, almost all participants lived in states with low stringency COVID-19 policies.


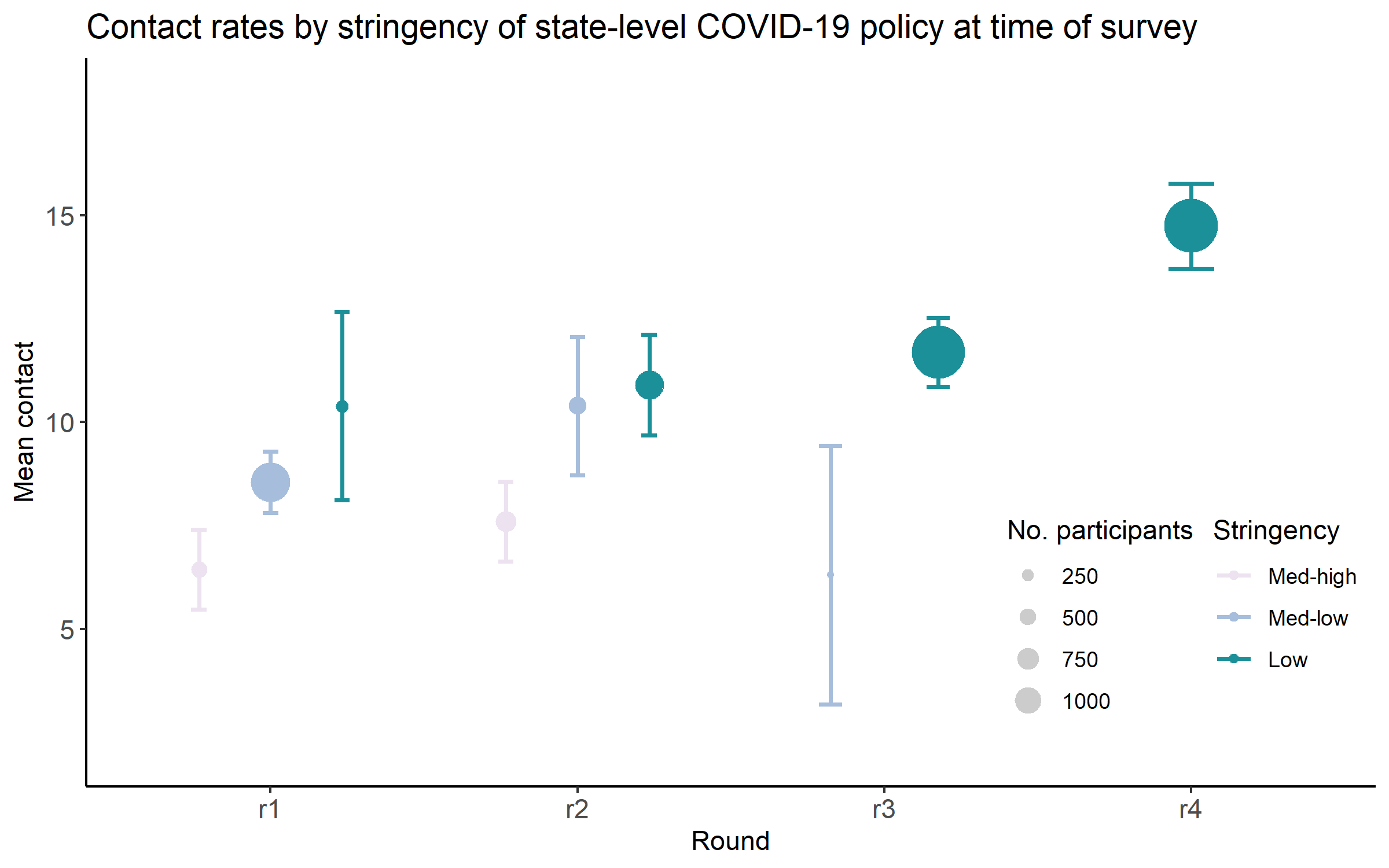


Figure S6. (Top) Mean contact rates over survey round by county-level vaccination coverage at time of survey. Across survey round, participants increasingly resided in counties with higher vaccination coverage, aligning with increasing vaccination from the start of vaccine rollout in January 2021 (round 2 data collection) and March 2022 (round 4 data collection). There were no clear patterns between mean contact rates and county-level vaccination coverage in rounds 2 and round 3 but by round 5, participants residing in areas with the highest vaccination coverage (>60% of total population) appeared to have the lowest mean contact. (Bottom) Vaccine coverage for primary vaccine series among the U.S. population over time.


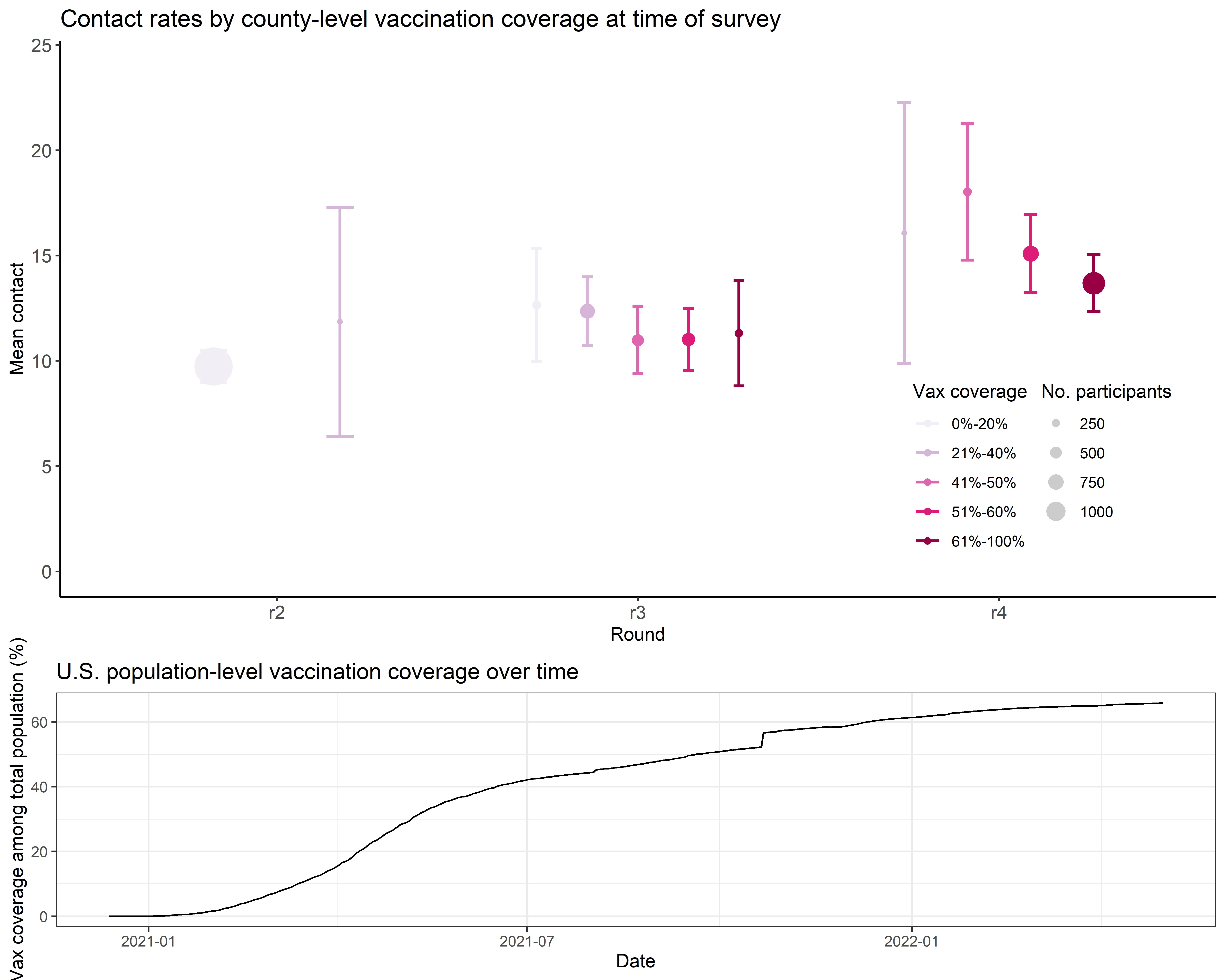


# SI.7 Effect of vaccination on changes in location-specific contact

Table S12. Effect of vaccination on changes in contact at work

| **Covariate** | **Category** | **Change in number of contacts** | |
| --- | --- | --- | --- |
|  |  | **Univariate associations** | **Multivariate associations** |
| **Intercept** |  |  | 5.05(0.59-9.51) |
| **Change in vaccination status** | Remain unvaxed |  |  |
|  | First dose new | 0.4(-1.32-2.12) | 0.83(-0.94-2.61) |
|  | Newly completed series | 0.19(-1.05-1.44) | 0.99(-0.4-2.39) |
|  | Already fully vaxed | 1.26(0.07-2.45) | 2.13(0.44-3.83) |
| **Every 20% increase in county-level vax coverage** | | 0.33(-0.03-0.68) | -0.26(-0.82-0.29) |
| **Age group** | 18-24 yrs |  |  |
|  | 25-34 yrs | -0.02(-2.39-2.35) | -0.26(-2.72-2.2) |
|  | 35-44 yrs | 0.34(-1.99-2.67) | -0.02(-2.45-2.4) |
|  | 45-54 yrs | -0.02(-2.34-2.3) | -0.12(-2.55-2.32) |
|  | 55-64 yrs | -0.7(-2.98-1.58) | -0.92(-3.29-1.46) |
|  | 65+ yrs | -1.44(-3.68-0.8) | -1.83(-4.23-0.57) |
| **Gender** | Female |  |  |
|  | Male | -0.23(-1.14-0.69) |  |
| **Race ethnicity** | Hispanic |  |  |
|  | Non-hispanic, White | -0.89(-2.3-0.52) |  |
|  | Non-hispanic, Black | -0.96(-2.77-0.85) |  |
|  | Non-hispanic, Asian | -1.5(-3.84-0.84) |  |
|  | Non-hispanic, Other | -1.97(-5.57-1.63) |  |
| **Household size** | 1 |  |  |
|  | 2-4 | -0.02(-1.04-0.99) | -0.25(-1.29-0.79) |
|  | 5+ | 1.53(-0.46-3.53) | 0.51(-1.62-2.63) |
| **Political affiliation** | Dem |  |  |
|  | Rep | -0.29(-1.6-1.02) | -0.45(-1.85-0.95) |
|  | Ind | 0.23(-1.01-1.47) | 0.36(-0.91-1.62) |
|  | Unknown | -0.59(-1.72-0.54) | -0.85(-2.05-0.34) |
| **Employment status** | Emp,in home |  |  |
|  | Emp,out home | -0.56(-1.79-0.66) | -0.47(-1.74-0.8) |
|  | Unemp | -1.5(-2.73--0.26) | -0.85(-2.25-0.54) |
|  | Unknown | 0.57(-1.93-3.07) | 0.16(-2.39-2.71) |
| **Household income** | $0-$24,999 |  |  |
|  | $25,000-$74,999 | 0.29(-1.35-1.93) |  |
|  | $75,000-$149,999 | 0.06(-1.6-1.71) |  |
|  | More than $150,000 | 0.25(-1.58-2.07) |  |
| **Comorbidities** | No |  |  |
|  | Yes | -0.18(-1.07-0.71) |  |
| **Risk tolerance (from Latent Class Analysis)** | High |  |  |
|  | Med-high | -0.46(-2.14-1.23) | -0.44(-2.23-1.34) |
|  | Med-low | -0.43(-2.11-1.25) | -0.23(-2.07-1.61) |
|  | Low | -0.88(-2.68-0.91) | -0.98(-2.88-0.92) |
| **Unit increase in baseline contact rates** | | -0.19(-0.22--0.15) |  |
| **Change in concern over pandemic** | Decreased greatly |  |  |
|  | Decreased slightly | -0.81(-4.14-2.53) | -1.03(-4.37-2.32) |
|  | No change | -2.32(-4.91-0.26) | -2.09(-4.7-0.51) |
|  | Increased slightly | -3.3(-5.87--0.73) | -3.14(-5.78--0.51) |
|  | Increased greatly | -2.87(-5.69--0.05) | -2.74(-5.64-0.16) |
| **Unit increase in state-wide Oxford Stringency Index** | | -0.03(-0.06-0.01) | 0(-0.05-0.05) |

Table S13. Effect of vaccination on changes in contact at other locations

| **Covariate** | **Category** | **Change in number of contacts** | |
| --- | --- | --- | --- |
|  |  | **Univariate associations** | **Multivariate associations** |
| **Intercept** |  | 0.2(0.02-0.38) | 0.09(-0.77-0.95) |
| **Change in vaccination status** | Remain unvaxed |  |  |
|  | First dose new | 0.22(-0.11-0.55) | 0.17(-0.17-0.52) |
|  | Newly completed series | -0.1(-0.33-0.14) | -0.02(-0.29-0.25) |
|  | Already fully vaxed | -0.17(-0.39-0.06) | -0.05(-0.38-0.27) |
| **Every 20% increase in county-level vax coverage** | | -0.07(-0.14-0) | 0(-0.11-0.11) |
| **Age group** | 18-24 yrs |  |  |
|  | 25-34 yrs | 0.18(-0.28-0.63) | 0.24(-0.24-0.71) |
|  | 35-44 yrs | 0.21(-0.23-0.66) | 0.27(-0.2-0.74) |
|  | 45-54 yrs | 0.13(-0.31-0.58) | 0.19(-0.27-0.66) |
|  | 55-64 yrs | 0.22(-0.21-0.66) | 0.27(-0.18-0.73) |
|  | 65+ yrs | 0.22(-0.21-0.65) | 0.25(-0.21-0.71) |
| **Gender** | Female |  |  |
|  | Male | 0.06(-0.11-0.24) |  |
| **Race ethnicity** | Hispanic |  |  |
|  | Non-hispanic, White | -0.13(-0.4-0.14) |  |
|  | Non-hispanic, Black | -0.23(-0.58-0.11) |  |
|  | Non-hispanic, Asian | -0.23(-0.68-0.22) |  |
|  | Non-hispanic, Other | 0.21(-0.48-0.9) |  |
| **Household size** | 1 |  |  |
|  | 2-4 | 0.01(-0.19-0.2) | 0(-0.2-0.2) |
|  | 5+ | 0.08(-0.3-0.47) | 0.09(-0.32-0.5) |
| **Political affiliation** | Dem |  |  |
|  | Rep | 0.07(-0.18-0.32) | 0(-0.27-0.27) |
|  | Ind | 0.11(-0.13-0.34) | 0.08(-0.16-0.33) |
|  | Unknown | 0.12(-0.1-0.34) | 0.07(-0.16-0.3) |
| **Employment status** | Emp,in home |  |  |
|  | Emp,out home | 0.14(-0.09-0.38) | 0.13(-0.11-0.38) |
|  | Unemp | 0.18(-0.06-0.41) | 0.2(-0.07-0.47) |
|  | Unknown | 0.16(-0.32-0.64) | 0.11(-0.38-0.6) |
| **Household income** | $0-$24,999 |  |  |
|  | $25,000-$74,999 | -0.02(-0.33-0.28) |  |
|  | $75,000-$149,999 | -0.09(-0.4-0.22) |  |
|  | More than $150,000 | -0.05(-0.38-0.29) |  |
| **Comorbidities** | No |  |  |
|  | Yes | -0.01(-0.18-0.16) |  |
| **Risk tolerance (from Latent Class Analysis)** | High |  |  |
|  | Med-high | -0.12(-0.44-0.2) | -0.08(-0.42-0.26) |
|  | Med-low | -0.21(-0.53-0.11) | -0.17(-0.53-0.18) |
|  | Low | -0.06(-0.41-0.28) | -0.04(-0.4-0.33) |
| **Unit increase in baseline contact rates** | | -0.19(-0.23--0.16) |  |
| **Change in concern over pandemic** | Decreased greatly |  |  |
|  | Decreased slightly | -0.36(-1-0.28) | -0.34(-0.99-0.3) |
|  | No change | -0.44(-0.93-0.06) | -0.51(-1.01-0) |
|  | Increased slightly | -0.39(-0.89-0.1) | -0.46(-0.96-0.05) |
|  | Increased greatly | -0.51(-1.05-0.03) | -0.56(-1.12--0.01) |
| **Unit increase in state-wide Oxford Stringency Index** | | 0.01(0-0.01) | 0.01(0-0.02) |

Table S14. Effect of vaccination on changes in contact at home

| **Covariate** | **Category** | **Change in number of contacts** | |
| --- | --- | --- | --- |
|  |  | **Univariate associations** | **Multivariate associations** |
| **Intercept** |  |  | 1.13(-1.39-3.65) |
| **Change in vaccination status** | Remain unvaxed |  |  |
|  | First dose new | 0.03(-0.94-1) | -0.01(-1.01-0.99) |
|  | Newly completed series | 1.02(0.32-1.72) | 0.94(0.16-1.73) |
|  | Already fully vaxed | 0.61(-0.05-1.28) | 0.5(-0.45-1.46) |
| **Every 20% increase in county-level vax coverage** | | 0.06(-0.14-0.26) | -0.14(-0.45-0.17) |
| **Age group** | 18-24 yrs |  |  |
|  | 25-34 yrs | 0.46(-0.87-1.79) | 0.48(-0.91-1.87) |
|  | 35-44 yrs | 0.85(-0.46-2.16) | 0.91(-0.45-2.28) |
|  | 45-54 yrs | 0.71(-0.6-2.01) | 0.89(-0.48-2.26) |
|  | 55-64 yrs | 0.9(-0.38-2.18) | 1.13(-0.21-2.47) |
|  | 65+ yrs | 1.49(0.23-2.75) | 1.58(0.22-2.93) |
| **Gender** | Female |  |  |
|  | Male | 0.08(-0.43-0.6) |  |
| **Race ethnicity** | Hispanic |  |  |
|  | Non-hispanic, White | 0.4(-0.39-1.19) |  |
|  | Non-hispanic, Black | 0.13(-0.89-1.15) |  |
|  | Non-hispanic, Asian | -0.32(-1.63-1) |  |
|  | Non-hispanic, Other | -0.08(-2.1-1.95) |  |
| **Household size** | 1 |  |  |
|  | 2-4 | 0.2(-0.37-0.78) | 0.37(-0.22-0.96) |
|  | 5+ | 0.42(-0.7-1.55) | 0.74(-0.46-1.94) |
| **Political affiliation** | Dem |  |  |
|  | Rep | 0.73(-0.01-1.47) | 0.42(-0.37-1.21) |
|  | Ind | 0.31(-0.39-1) | 0.22(-0.49-0.93) |
|  | Unknown | 0.39(-0.24-1.03) | 0.44(-0.24-1.11) |
| **Employment status** | Emp,in home | 0.46(-0.1-1.03) |  |
|  | Emp,out home | 0.08(-0.61-0.77) | -0.04(-0.75-0.68) |
|  | Unemp | 0.45(-0.25-1.14) | 0(-0.79-0.79) |
|  | Unknown | 0.4(-1.01-1.8) | 0.23(-1.21-1.66) |
| **Household income** | $0-$24,999 | 0.42(-0.36-1.2) |  |
|  | $25,000-$74,999 | 0.24(-0.66-1.14) |  |
|  | $75,000-$149,999 | 0.31(-0.6-1.23) |  |
|  | More than $150,000 | 0.48(-0.53-1.49) |  |
| **Comorbidities** | No | 0.62(0.26-0.98) |  |
|  | Yes | 0.1(-0.39-0.6) |  |
| **Risk tolerance (from Latent Class Analysis)** | High | 1.23(0.38-2.08) |  |
|  | Med-high | -0.74(-1.69-0.2) | -0.8(-1.81-0.21) |
|  | Med-low | -0.46(-1.4-0.48) | -0.66(-1.7-0.38) |
|  | Low | -0.63(-1.64-0.37) | -0.66(-1.73-0.41) |
| **Unit increase in baseline contact rates** | | -0.21(-0.25--0.16) |  |
| **Change in concern over pandemic** | Decreased greatly | 1.82(0.42-3.22) |  |
|  | Decreased slightly | -1.06(-2.94-0.83) | -0.92(-2.8-0.97) |
|  | No change | -1.11(-2.57-0.36) | -1.1(-2.57-0.37) |
|  | Increased slightly | -1.33(-2.79-0.12) | -1.23(-2.72-0.26) |
|  | Increased greatly | -0.7(-2.3-0.89) | -0.65(-2.28-0.99) |
| **Unit increase in state-wide Oxford Stringency Index** | | -0.02(-0.03-0) | -0.02(-0.04-0.01) |

# SI.8 Effect of vaccination on changes in all contacts without concern for new variants

Table S15. Effect of vaccination on changes in all contacts without concern for new variants.

| **Covariate** | **Category** | **Change in number of contacts** | |
| --- | --- | --- | --- |
|  |  | **Univariate associations** | **Multivariate associations** |
| **Intercept** |  |  | 2.44(-1.91-6.78) |
| **Change in vaccination status** | Remain unvaxed |  |  |
|  | First dose new | 0.59(-1.46-2.64) | 1.08(-1.01-3.17) |
|  | Newly completed series | 1.11(-0.37-2.58) | 1.66(0.03-3.3) |
|  | Already fully vaxed | 1.9(0.48-3.31) | 2.69(0.69-4.7) |
| **Every 20% increase in county-level vax coverage** | | 1.4(0.98-1.82) | -0.01(-0.07-0.05) |
| **Age group** | 18-24 yrs |  |  |
|  | 25-34 yrs | 0.1(-2.71-2.92) | 0.05(-2.81-2.92) |
|  | 35-44 yrs | 0.88(-1.88-3.65) | 0.64(-2.19-3.46) |
|  | 45-54 yrs | 0.26(-2.5-3.02) | 0.24(-2.59-3.08) |
|  | 55-64 yrs | -0.15(-2.86-2.56) | -0.09(-2.87-2.68) |
|  | 65+ yrs | -0.39(-3.06-2.27) | -0.6(-3.41-2.21) |
| **Gender** | Female |  |  |
|  | Male | -0.08(-1.16-1.01) |  |
| **Race ethnicity** | Hispanic |  |  |
|  | Non-hispanic, White | -0.67(-2.35-1.01) |  |
|  | Non-hispanic, Black | -1.13(-3.29-1.02) |  |
|  | Non-hispanic, Asian | -1.95(-4.72-0.83) |  |
|  | Non-hispanic, Other | -2.01(-6.28-2.27) |  |
| **Household size** | 1 |  |  |
|  | 4-Feb | 0.25(-0.95-1.46) | 0.16(-1.08-1.39) |
|  | 5+ | 2.4(0.03-4.77) | 2.18(-0.31-4.67) |
| **Political affiliation** | Dem |  |  |
|  | Rep | 0.47(-1.09-2.03) | 0.56(-1.09-2.21)) |
|  | Ind | 0.67(-0.8-2.15) | 0.82(-0.68-2.31) |
|  | Unknown | 0.47(-1.09-2.03) | 0.15(-1.25-1.55) |
| **Employment status** | Emp,in home |  |  |
|  | Emp,out home | -0.33(-1.78-1.13) | -0.53(-2.04-0.97) |
|  | Unemp | -0.91(-2.38-0.56) | -0.79(-2.44-0.87) |
|  | Unknown | 1.07(-1.9-4.04) | 1.07(-1.94-4.08) |
| **Household income** | $0-$24,999 |  |  |
|  | $25,000-$74,999 | 0.44(-1.5-2.37) |  |
|  | $75,000-$149,999 | 0.16(-1.8-2.12) |  |
|  | More than $150,000 | 0.55(-1.61-2.71) |  |
| **Comorbidities** | No |  |  |
|  | Yes | -0.18(-1.23-0.88) |  |
| **Risk tolerance (from Latent Class Analysis)** | High |  |  |
|  | Med-high | -1.35(-3.35-0.65) | -1.6(-3.69-0.49) |
|  | Med-low | -1.08(-3.08-0.92) | -1.25(-3.4-0.9) |
|  | Low | -1.61(-3.74-0.53) | -1.85(-4.06-0.37) |
| **Unit increase in baseline contact rates** | | -0.16(-0.2--0.13) |  |
| **Change in concern over pandemic** | Increased greatly |  |  |
|  | Increased slightly | -2.3(-6.25-1.66) |  |
|  | No change | -4.07(-7.14--1) |  |
|  | Decreased slightly | -5.35(-8.4--2.3) |  |
|  | Decreased greatly | -4.46(-7.82--1.11) |  |
| **Unit increase in state-wide Oxford Stringency Index** | | -0.04(-0.08-0) | -0.17(-0.82-0.48) |

# SI.9 Sensitivity analysis on contact outlier cutoff

While we used the 99^th^ percentile to right truncate responses on number of contacts per location and age group, previous studies chose 100^18,34^ or 450 contacts as the cutoff^25^. We chose the 99^th^ percentile for our main analysis to remove only the most extreme outliers that were least likely to be accurate and to increase the absolute truncation thresholds across the rounds as social distancing relaxed and individuals were more likely to truly have high numbers of contact. For sensitivity analysis, we considered other truncation criteria of 1) 95^th^ percentile; 2) 97.5^th^ percentile and 3) 100 contacts per location. The truncation thresholds used for each round and location can be found in table below). Results for the main model with sensitivity analysis on choice of right truncation and be found in Figure S7.

Table S16. Cutoff values for various right truncation methods to remove extreme outlier responses in contact numbers that are unlikely to be accurate

|  |  | **Truncation value for various percentiles** | | | **No. reporting more than 100 contacts** |
| --- | --- | --- | --- | --- | --- |
| **Survey round** | **Contact location** | **95th** | **97.5th** | **99th** |  |
| Round 1 | Home | 6 | 10 | 13.98 | 2 |
| Round 1 | Other | 11 | 19 | 35 | 4 |
| Round 1 | School | 0 | 0 | 5 | 0 |
| Round 1 | Work | 23 | 45 | 89.96 | 21 |
| Round 2 | Home | 7 | 10 | 22.98 | 1 |
| Round 2 | Other | 12 | 21 | 47.98 | 7 |
| Round 2 | School | 0 | 0 | 6.98 | 2 |
| Round 2 | Work | 26.9 | 49.9 | 116.92 | 28 |
| Round 3 | Home | 7 | 10 | 19.98 | 1 |
| Round 3 | Other | 18 | 29.9 | 66.92 | 12 |
| Round 3 | School | 0 | 0 | 4 | 1 |
| Round 3 | Work | 30 | 54.95 | 119.9 | 30 |
| Round 4 | Home | 8 | 10 | 22.96 | 5 |
| Round 4 | Other | 20 | 34 | 73.96 | 17 |
| Round 4 | School | 0 | 5 | 23.98 | 6 |
| Round 4 | Work | 42.9 | 77 | 142.98 | 43 |

Sensitivity analysis with varying values of right truncation to remove extreme and unrealistic outliers of contacts reported by participants

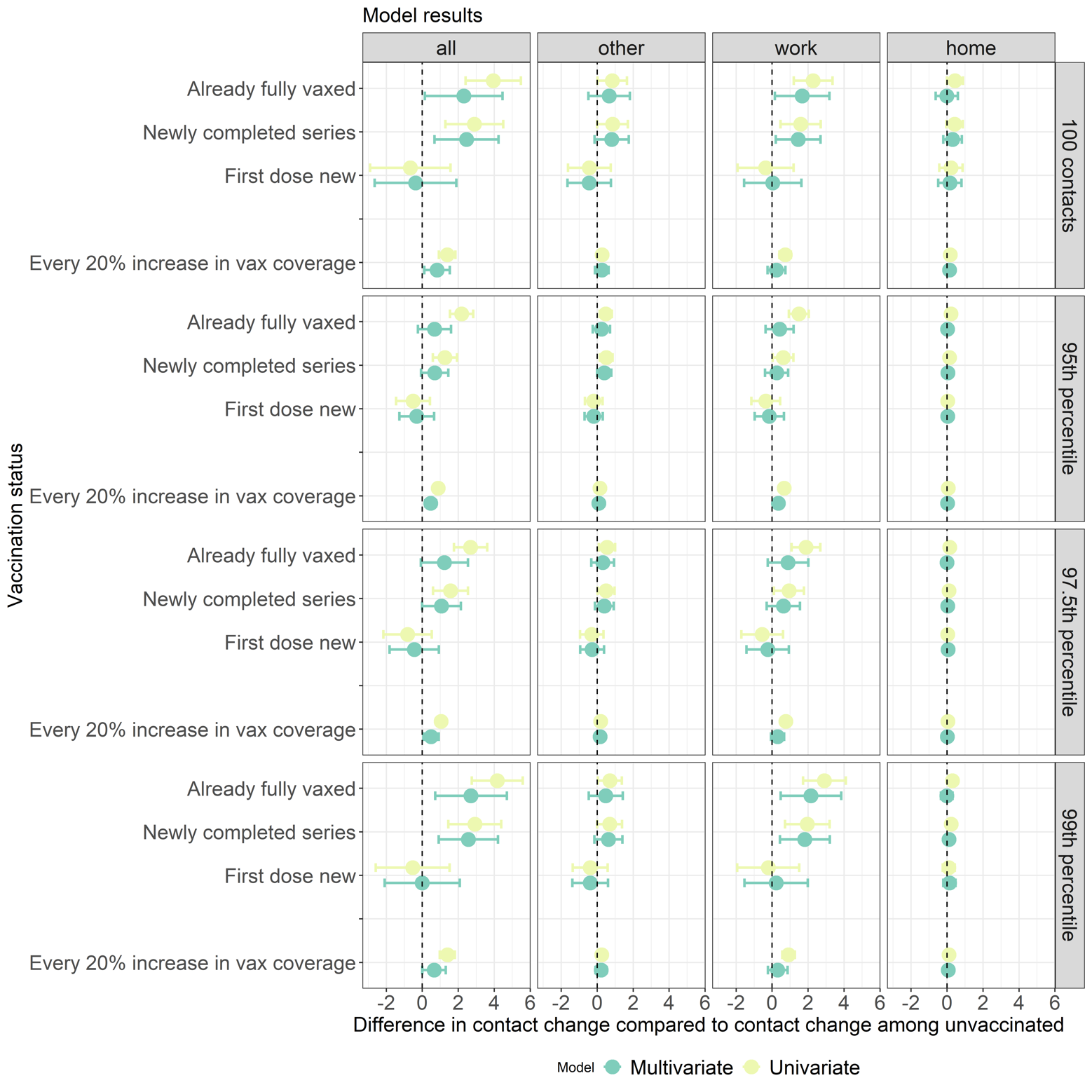


Figure S7. Plot of model results for the main analysis comparing different right truncation choices.

# SI.10 Additional Next Generation Matrix analysis on impact on transmission

For each level of vaccination coverage and for each round, we perturb the Next Generation Matrix by allowing for increasingly assortative mixing where vaccinated individuals mix more preferentially with other vaccinated individuals and unvaccinated individuals mix more preferentially with other unvaccinated individuals. We quantify the extent of assortativity with the Q index, which is calculated as Q = [Tr(P)-1]/(n-1), where P is a matrix with elements of $P_{ij}= M_{ij}/\sum_{j} M_{ij}$ is the matrix of average contacts between vax groups^43,44^. Tr(P) is the trace of the matrix or the sum of its diagonals and n is the number of subgroups (n=2). The Q index is 0 when mixing between subgroups is completely proportional and 1 when mixing is completely assortative (vaccinated individuals only contact other vaccinated individuals).


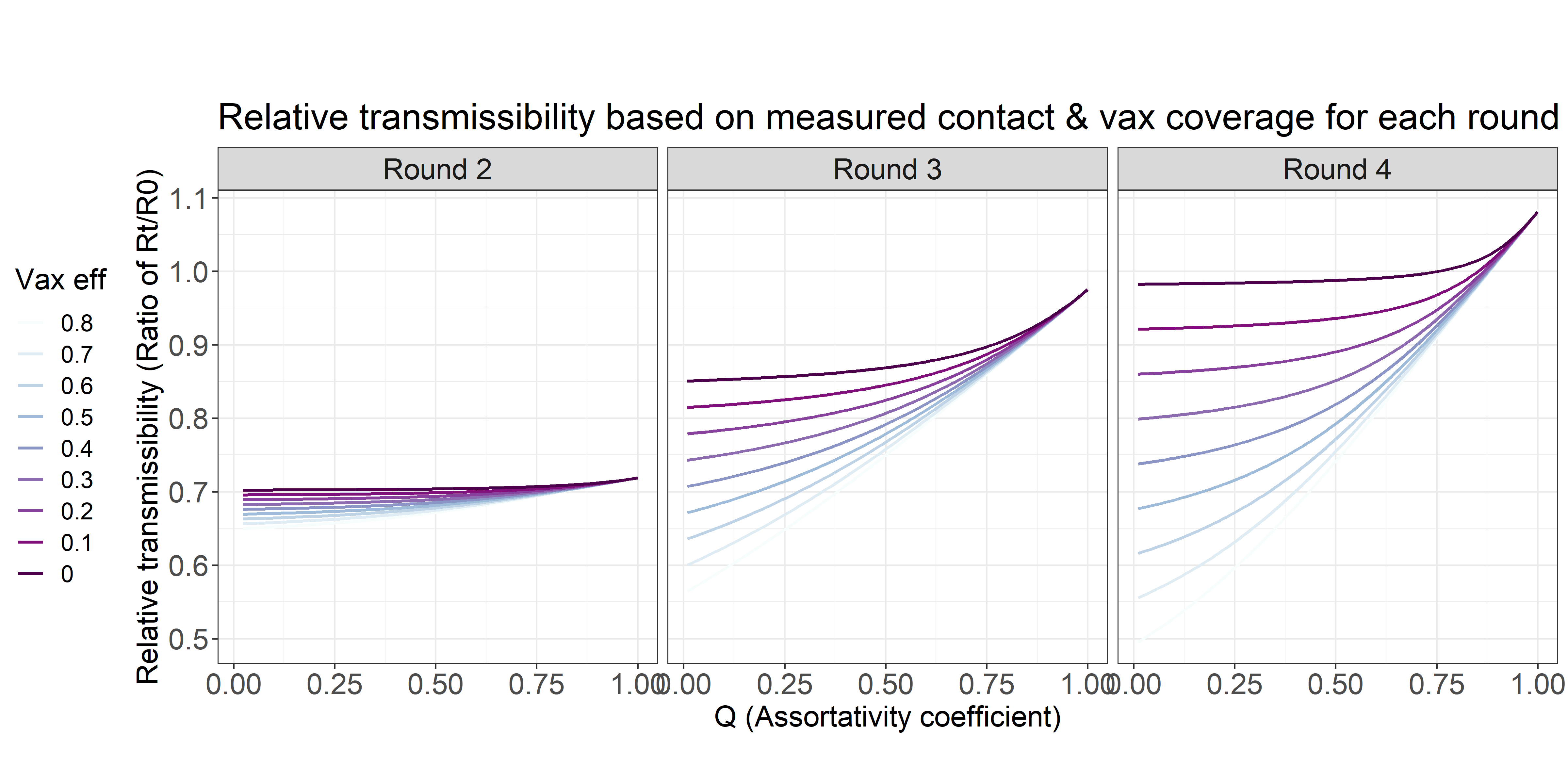


Figure S8. Relative transmissibility (Rt/R0) calculated using the Next Generation Matrix (see methods section in main paper) based on measured contact in vaccinated and unvaccinated individuals in each round and overall vaccine coverage in the US, sweeping over a range of vaccine effectiveness against susceptibility and assortativity coefficient (Q=0 for proportional mixing based on vaccine coverage, Q=1 for assortative mixing where vaccinated individuals only mix with other vaccinated individuals and unvaccinated individuals only mix with other unvaccinated individuals). From round 2 (March-April, 2021) to round 4 (March-April, 2022), the average daily contact rates among vaccinated individuals were 9.2, 11.6 and 14.9; average daily contact rates among unvaccinated individuals were 11.5, 15.6, 17.3 and the primary series vaccine coverage in the U.S. was 11.5%, 49.8% and 65.9%. As contact rates increased across survey rounds, relative transmissibility increased even as more individuals in the population became vaccinated. We find the relative transmissibility increases as the population mixes more assortatively (increasing Q values) and that with fully assortative mixing (Q=1), the relative transmissibility converges regardless of the vaccine effectiveness.
